# An intrinsic mechanism for coordinated production of the contact-dependent and contact-independent weapon systems in a soil bacterium

**DOI:** 10.1371/journal.ppat.1008967

**Published:** 2020-10-09

**Authors:** Mingming Yang, Shuangshuang Ren, Danyu Shen, Nianda Yang, Bingxin Wang, Sen Han, Xi Shen, Shan-Ho Chou, Guoliang Qian

**Affiliations:** 1 College of Plant Protection (Laboratory of Plant Immunity, Key Laboratory of Integrated Management of Crop Diseases and Pests), Nanjing Agricultural University, Nanjing, P.R. China; 2 Institute of Biochemistry, and NCHU Agricultural Biotechnology Center, National Chung Hsing University, Taichung, ROC, Taiwan; Academia Sinica, TAIWAN

## Abstract

Soil bacteria possess multiple weapons to fend off microbial competitors. Currently, we poorly understand the factors guiding bacterial decisions about weapon systems deployment. In this study, we investigated how such decisions are made by the soil bacterium *Lysobacter enzymogenes*, used in antifungal plant protection. We found that weapons production is guided by environmental cues. In rich media, which likely mimic environments crowded with other microbes, *L*. *enzymogenes* produces a contact-dependent weapon, type six secretion system (T6SS). In nutrient-poor media, likely dominated by filamentous oomycetes and fungi, *L*. *enzymogenes* synthesizes and secretes a heat-stable antifungal factor (HSAF), a contact-independent weapon. Surprisingly, the T6SS inner tube protein Hcp is accumulated intracellularly even in nutrient-poor media, when the T6SS is not assembled. We found that Hcp interacts with the transcription factor Clp required for activating HSAF biosynthesis operon expression. Hcp protects Clp from binding to c-di-GMP, an intracellular second messenger inhibiting DNA binding. The increased concentration of c-di-GMP-free Clp thus leads to higher gene expression and HSAF production. Therefore, when the contact-dependent weapon, T6SS, is not in use, accumulation of one of its structural components, Hcp, serves as a signal to enhance production of the contact-independent weapon, HSAF. The uncovered environment-dependent and auto-regulatory mechanisms shed light on the processes governing deployment of various weapon systems in environmental bacteria.

## Author summary

Soil bacteria face competition from diverse microbial species. To stay competitive, they deploy a variety of weapons. At present, we know little about factors influencing decisions about which weapons to produce at any given time, and about mechanisms through which these decisions are carried out. In this study, we show that in the soil bacterium, *Lysobacter enzymogenes*, synthesis of the contact-dependent weapon, known as type six secretion system (T6SS) occurs under different conditions, compared to those conductive to the production of the contact-independent weapon, toxin HSAF. Further, when T6SS is not assembled, one of its structural components, Hcp, coactivates HSAF operon expression and HSAF synthesis. This study reveals that decisions about contact-dependent and contact-independent weapon production in bacteria are governed by both environmental cues and intrinsic coordination mechanisms.

## Introduction

Soil bacteria are surrounded by diverse competitors that include other bacteria, fungi, oomycetes and protists [1, 2]. To fend off these competitors, bacteria deploy various toxins, which are either secreted into the surrounding medium (contact-independent weapons) or injected directly into the competitor cell (contact-dependent weapons). Type six secretion system (T6SS) is a form of a contact-dependent weapon resembling a nano-speargun evolved from a contractile phage tail [3]. T6SS is commonly used by the proteobacteria for contact-induced killing. Upon cell-cell contact, an engaged T6SS injects a toxin-loaded shell, known as inner tube, into the target cell by piercing though its cell wall and membranes [4, 5]. T6SS toxins possess diverse killing modalities that are effective primarily against bacteria [6–8]. However, some reports documented the use of the T6SS by the Gram-negative bacteria against fungi. For example, *Serratia marcescens*, delivers antifungal effectors against *Saccharomyces cerevisiae* and pathogenic *Candida albicans* [9, 10].

Diffusible toxins are the main bacterial weapon against oomycetes and fungi [11]. Bacteria of the genus *Lysobacter* are renown for killing filamentous oomycetes and fungi by secreting toxins that freely diffuse into the surrounding medium [12, 13]. *Lysobacter enzymogenes*, the best characterized species of the genus, secretes a broad-spectrum toxin, known as heat-stable antifungal factor (HSAF). The mechanism of HSAF action involves disrupting fungal sphingolipid biosynthesis and inducing apoptosis [14, 15]. It is believed that HSAF not only helps *L*. *enzymogenes* fight oomycetes and fungi but also allows it to prey on these cells. The ability of *L*. *enzymogenes* to effectively kill plant pathogenic oomycetes and fungi makes it an attractive natural crop protecting agent [16, 17].

Our earlier studies have established that HSAF secretion is high in nutrient-poor media and low in rich media [18, 19]. The HSAF production is primarily regulated at the expression level of the HSAF biosynthesis operon, which is controlled by several factors. Clp is the dominant transcription activator [20]. It binds to two sites (PA and PB) upstream of the promoter of the HSAF biosynthesis operon and activates gene expression and HSAF production [21]. Clp can also bind the second messenger c-di-GMP, which decreases its DNA binding ability and thus lowers HSAF operon gene expression [21]. LchP, the main c-di-GMP degrading phosphodiesterase in *L*. *enzymogenes*, forms a signaling complex with Clp [21]. Inactivation of the *lchP* gene results in higher cellular c-di-GMP levels and lower HSAF production [21].

In this work, we intended to better understand factors affecting deployment of various weapon systems in *L*. *enzymogenes*. Indiscriminate weapons overproduction can be burdensome for growth. The need to adjust weapon systems deployment dependent on the surroundings is intuitive. Indeed, we found that T6SS and HSAF are produced under different nutrient conditions, where the density and type of microbial competitors of *L*. *enzymogenes* likely differ. We were, however, intrigued by the unexpected observation that when HSAF was produced, one component of T6SS, the inner tube protein Hcp, continued to be synthesized. We found that the intracellularly accumulated Hcp assumes a second, regulatory role as a Clp-coactivator. Thus Hcp, a structural component of the contact-dependent killing machine, T6SS, activates the production of the contact-independent weapon HSAF, when T6SS is not needed. This ingenious mechanism represents an example of intrinsic coordination in the production of the contact-dependent and contact-independent weapons in a soil bacterium.

## Results

### T6SS and HSAF are synthesized under different conditions

**Fig 1:**
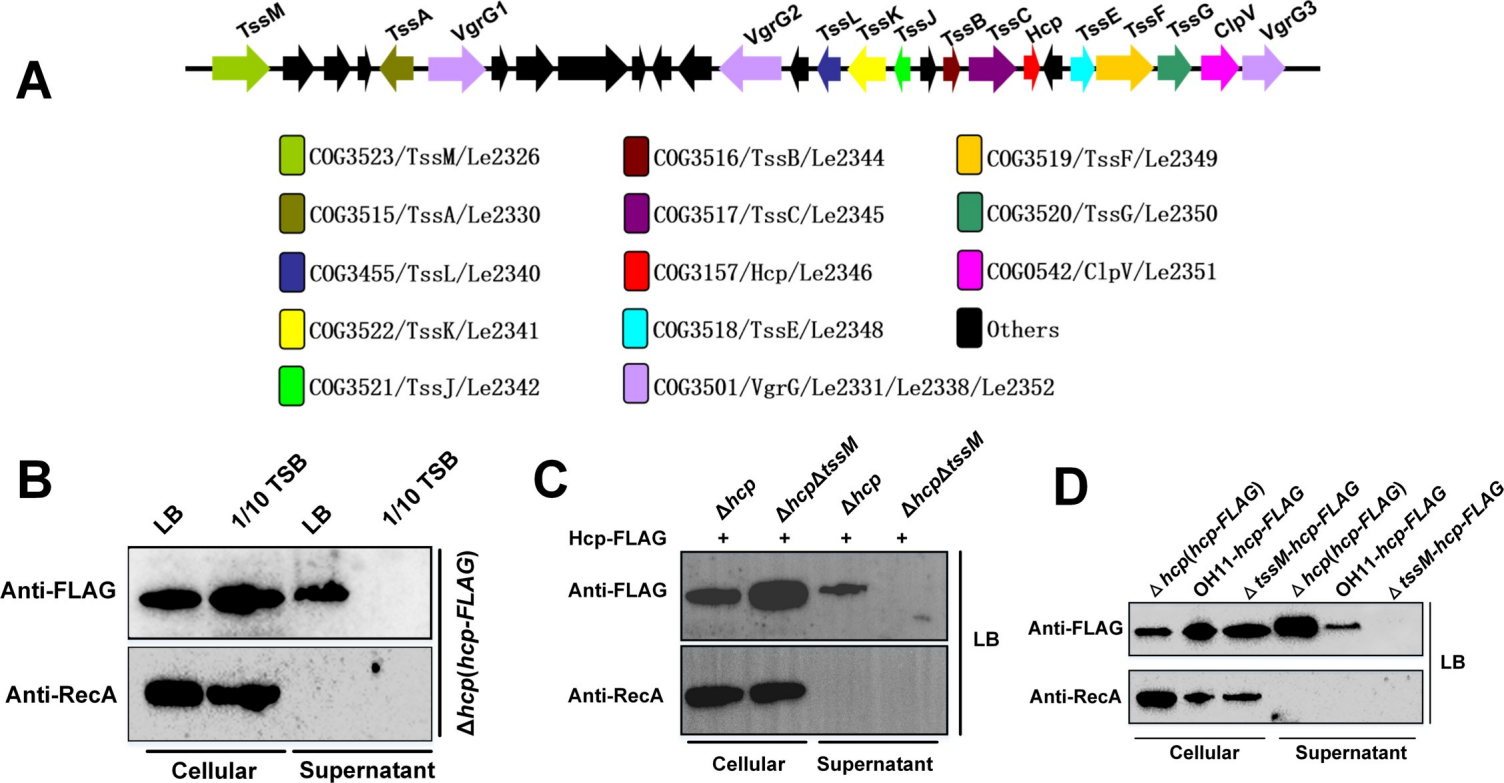
A functional T6SS system is assembled in *L*. *enzymogenes* grown in rich medium. **(A)** The *L*. *enzymogenes* T6SS gene cluster. The genes encoding T6SS components, identified with the help of the database of Clusters of Orthologous Groups of proteins (COGs) [25, 40], are shown in color. **(B)** Western blot showing that Hcp is secreted by the Δ*hcp* mutant expressing the plasmid-borne Hcp-FLAG, when the strain is grown in rich (LB) but not nutrient-poor (1/10 TSB) medium. The housekeeping protein RecA was used as intracellular control. **(C)** Western blot revealing that the *tssM* deletion blocks the secretion of the plasmid-expressed Hcp-FLAG in the strains grown in LB. **(D)** Western blot showing that the *tssM* deletion impairs the secretion of the chromosomally expressed Hcp-FLAG. The assays were repeated three times with similar results. OH11, wild-type strain; Δ*hcp*, the *hcp* deletion mutant; Δ*hcp* Δ*tssM*, the *tssM* and *hcp* double mutant; (*hcp-FLAG*), the plasmid-borne *hcp-FLAG* gene expressed from the native *hcp* promoter; OH11-*hcp-FLAG*, the OH11 derivative strain, in which the chromosomal *hcp* gene was replaced with the *hcp-FLAG* allele; Δ*tssM*-*hcp-FLAG*, the Δ*tssM* mutant, in which the chromosomal *hcp* gene was replaced with the *hcp-FLAG*. Anti-FLAG, monoclonal antibody against FLAG; Anti-RecA, monoclonal antibody against RecA.

The *L*. *enzymogenes* OH11 genome contains a T6SS gene cluster (Fig 1A and S1 Table), and our earlier transcriptomics study revealed that the T6SS genes are transcribed under the HSAF-producing conditions, in nutrient-poor medium, 1/10 TSB [22]. However, it remained unknown whether or not a functional T6SS was assembled under these conditions. To test for assembly of a functional T6SS, we relied on a previously described assay that involves the detection in the culture medium of Hcp, the inner tube protein of the T6SS Hcp can be secreted in the growth medium only via the assembled T6SS [23–25].

For Hcp detection, we generated an Hcp fusion with the FLAG-tag and expressed the *hcp-FLAG* gene from a plasmid under the native *hcp* promoter in the *L*. *enzymogenes hcp* chromosomal mutant, Δ*hcp* (S2 Table). We grew this strain in 1/10 TSB, under the same conditions under which the T6SS gene cluster transcripts were previously detected [22]. To our surprise, the Hcp-FLAG protein was undetectable in the culture supernatant (Fig 1B). This observation suggests that T6SS is not assembled in the nutrient-poor medium. Intriguingly, under these conditions, the *hcp* gene was not only transcribed, but translated its product, the Hcp protein that was accumulated intracellularly (Fig 1B). We shall address this observation later in this study.

In search of conditions under which T6SS is assembled, we switched to rich medium, LB [22]. The plasmid-expressed Hcp-FLAG was readily detectable in the culture supernatant of the strain grown in LB (Fig 1B). To ensure that Hcp was secreted through the T6SS, we tested whether or not it is secreted in a mutant impaired in T6SS assembly. To this end, we deleted the chromosomal *tssM* gene encoding a structural component of the T6SS membrane-anchoring complex [25] in the Δ*hcp* background. In the Δ*hcp* Δ*tssM* double mutant containing the plasmid-encoded *hcp-FLAG*, no secreted Hcp-FLAG protein was detected in the supernatant (Fig 1C), consistent with the assumption that Hcp-FLAG is only secreted through the assembled T6SS. Further supporting this conclusion are the following observations. (i) The abundance of intracellular, chromosomally expressed Hcp-FLAG was similar in the Δ*hcp* and Δ*hcp* Δ*tssM* strains expressing *hcp-FLAG*, whereas secretion was observed only in the Δ*hcp* strain (Fig 1C and 1D). (ii) Hcp-FLAG did not leak into the medium via cell lysis, because the levels of the housekeeping protein, RecA [26, 27], in culture supernatants were below detection (Fig 1B–1D).

### The T6SS inner tube protein, Hcp, is required for full-scale HSAF production

**Fig 2:**
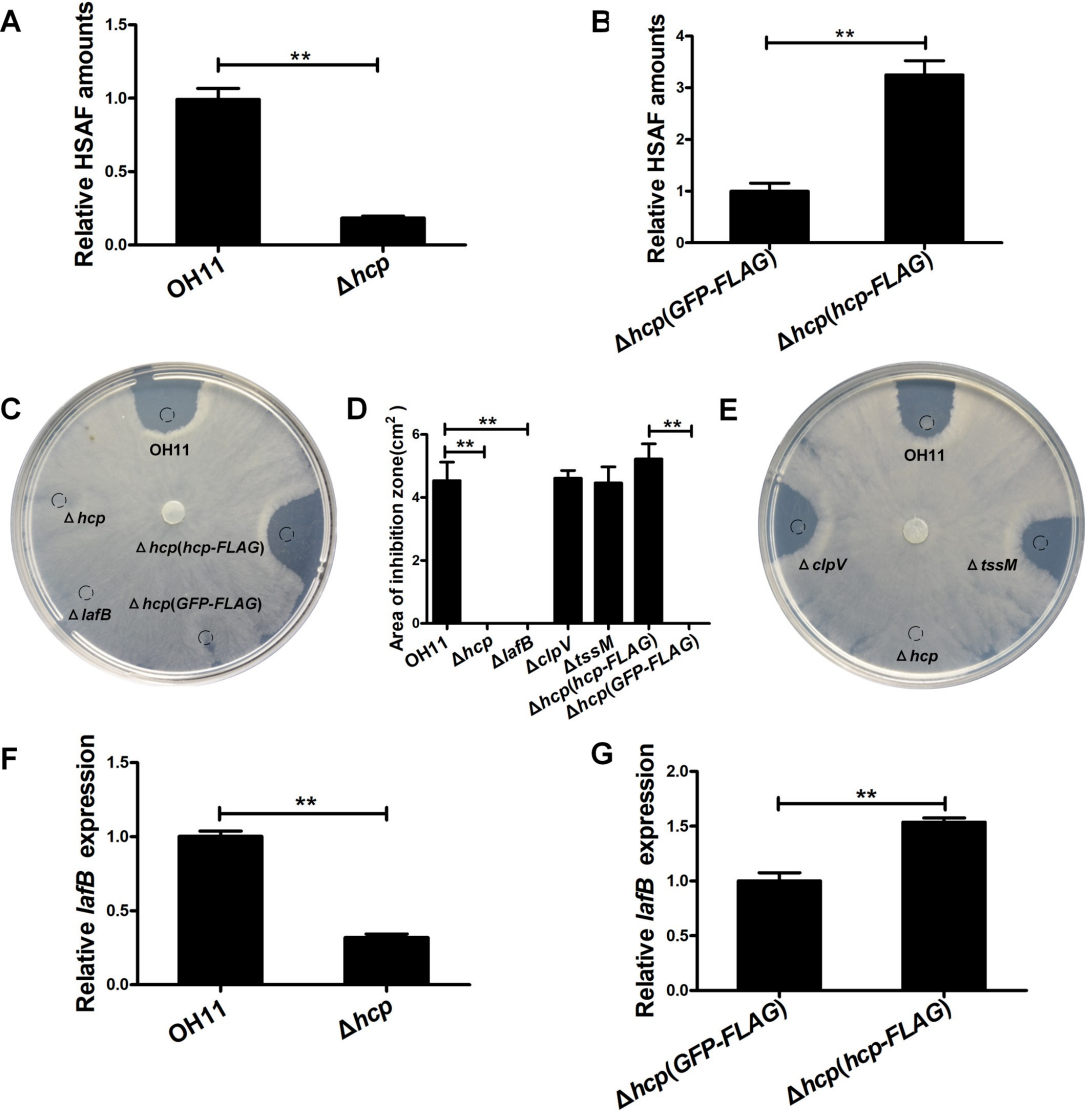
Hcp contributes to growth inhibition of *P*. *capsica* by regulating HSAF production in *L*. *enzymogenes*. **(A-B)** Relative HSAF production. Bacteria were grown in 1/10 TSB without gentamycin—strains OH11 and Δ*hcp* (**A**), or with gentamycin—strains containing plasmids expressing either *GFP-FLAG* and *hcp-*FLAG (**B**). **(C-E)** Plating assay showing inhibition of *P*. *capsici* growth by the HSAF extracts from *L*. *enzymogenes* (**C, E**), and statistical analysis of inhibition zones (**D**). Dash-lined circles indicate the sites of HSAF spotting. Lengths of the longest axis and shortest axis of the inhibition zones were determined and averaged as the radius, R, according to an earlier report [33]. The inhibition zones were calculated by using the formula π × R^2^. Average data from three experiments are presented, ± SD. **P < 0.01. (**F-G**) Relative *lafB* mRNA abundance measured by qRT-PCR in the Δ*hcp* mutant and wild-type, OH11, grown in 1/10 TSB without gentamycin (**F**), and in the Δ*hcp* mutant containing the plasmid-expressed *GFP-FLAG* and *hcp-FLAG*, grown in 1/10 TSB with gentamycin (**G**). Average data from three experiments are shown, ± SD. **P < 0.01. For strain description, see legend to Fig 1.

We detected no indications that the FLAG-tag on Hcp interferes with *L*. *enzymogenes* physiology. For example, HSAF production in the wild-type strain OH11 containing the chromosomal *hcp*-*FLAG* allele in place of the *hcp* gene (designated OH11-*hcp*-*FLAG*) did not differ from the HSAF production in the wild type, OH11, as judged by growth inhibition of the filamentous oomycete *Phytophthora capsica* (S1 Fig). The Δ*hcp* mutant displayed no growth defects either, compared to the wild type (S2 Fig). Unexpectedly, the amount of HSAF produced by this mutant was only approximately thirty percent of the wild-type amount (Fig 2A and S3 Fig). Introduction of the plasmid-borne *hcp*-*FLAG* in the Δ*hcp* mutant significantly increased the HSAF yield (Fig 2B and S3 Fig), confirming that lower HSAF production was caused by the absence of Hcp.

Consistent with HSAF measurements, the HSAF extracts from the Δ*hcp* mutant failed to inhibit *P*. *capsici* growth in a plating assay, in contrast to the HSAF extract from the wild type (Fig 2C and 2D). Introduction in the Δ*hcp* mutant of the plasmid-borne *hcp*-*FLAG*, but not *GFP-FLAG* used as negative control, rescued this deficiency. To verify that inhibition of the *P*. *capsici* growth was due to HSAF, we used the extract from a HSAF-deficient strain that contains an in-frame deletion in *lafB*, the first gene of the HSAF biosynthetic operon. As expected, this HSAF-deficient strain failed to inhibit *P*. *capsici* growth (Fig 2C and 2D). The unexplained observation that the T6SS component, Hcp, is required for the full-scale HSAF production under the conditions where T6SS is not assembled, encouraged us to investigate the mechanism linking Hcp and HSAF.

### Regulatory function of the Hcp protein

First, we tested whether the effect of Hcp on HSAF levels was protein-specific or common among the T6SS structural components. To this end, we generated mutations in several T6SS genes, i.e. *clpV*, *tssM*, *vgrG2* and *vgrG3* (Fig 1A). Mutations in none of these genes reduced HSAF levels (S4A Fig). The HSAF extracts from two representative T6SS-deficient mutants, Δ*tssM* and Δ*clpV*, inhibited *P*. *capsici* in the plating assay (Fig 2D and 2E). Therefore, it appears that unlike other T6SS components, Hcp plays a specific role in HSAF production. To further confirm the specificity of the Hcp-HSAF connection, we performed a series of additional phenotypic tests, including colony morphology (S4B Fig), production of an extracellular chitinase (S4C Fig), and twitching motility (S4D Fig and S4E Fig), using the generated T6SS gene mutants. No obvious defects were observed, compared to the wild type, suggesting that the effect of Hcp is specific to HSAF production.

Next, we investigated the mechanism through which Hcp affects HSAF production. Since the amount of secreted HSAF is primarily controlled at the expression level of the HSAF biosynthesis operon [16, 28], we checked whether Hcp is involved in gene regulation. We measured the mRNA abundance of the first gene *lafB* in the HSAF biosynthesis operon, and found that in the Δ*hcp* mutant, it was reduced by approximately sixty-five percent, compared to the wild type. Introduction of the plasmid-borne *hcp*-*FLAG*, but not *GFP-FLAG*, in the Δ*hcp* mutant drastically increased the *lafB* transcript abundance (Fig 2F and 2G). These results collectively suggest that the intracellular Hcp regulates, directly or indirectly, expression of the HSAF biosynthesis operon.

### Hcp interacts with Clp, the key transcription activator of the HSAF biosynthesis operon

Since Hcp lacks a detectable DNA-binding domain, we hypothesized that it controls the HSAF biosynthesis operon expression via a transcription factor. To identify such a factor, we carried out a co-immunoprecipitation (co-IP) assay coupled with mass spectrometry. In this assay, Hcp-FLAG was used as a bait. Several potential Hcp-FLAG-interacting proteins were identified (S2 Table), including Hcp itself. Since Hcp-Hcp interactions are important for inner tube assembly, this finding served as a positive control. The majority of other interactors were highly abundant cellular proteins, e.g. ribosomal proteins and chaperons, that may bind Hcp in a nonspecific manner. Interestingly, among the potential Hcp interactors was Clp, the key transcription activator of the HSAF biosynthesis operon [21].

**Fig 3:**
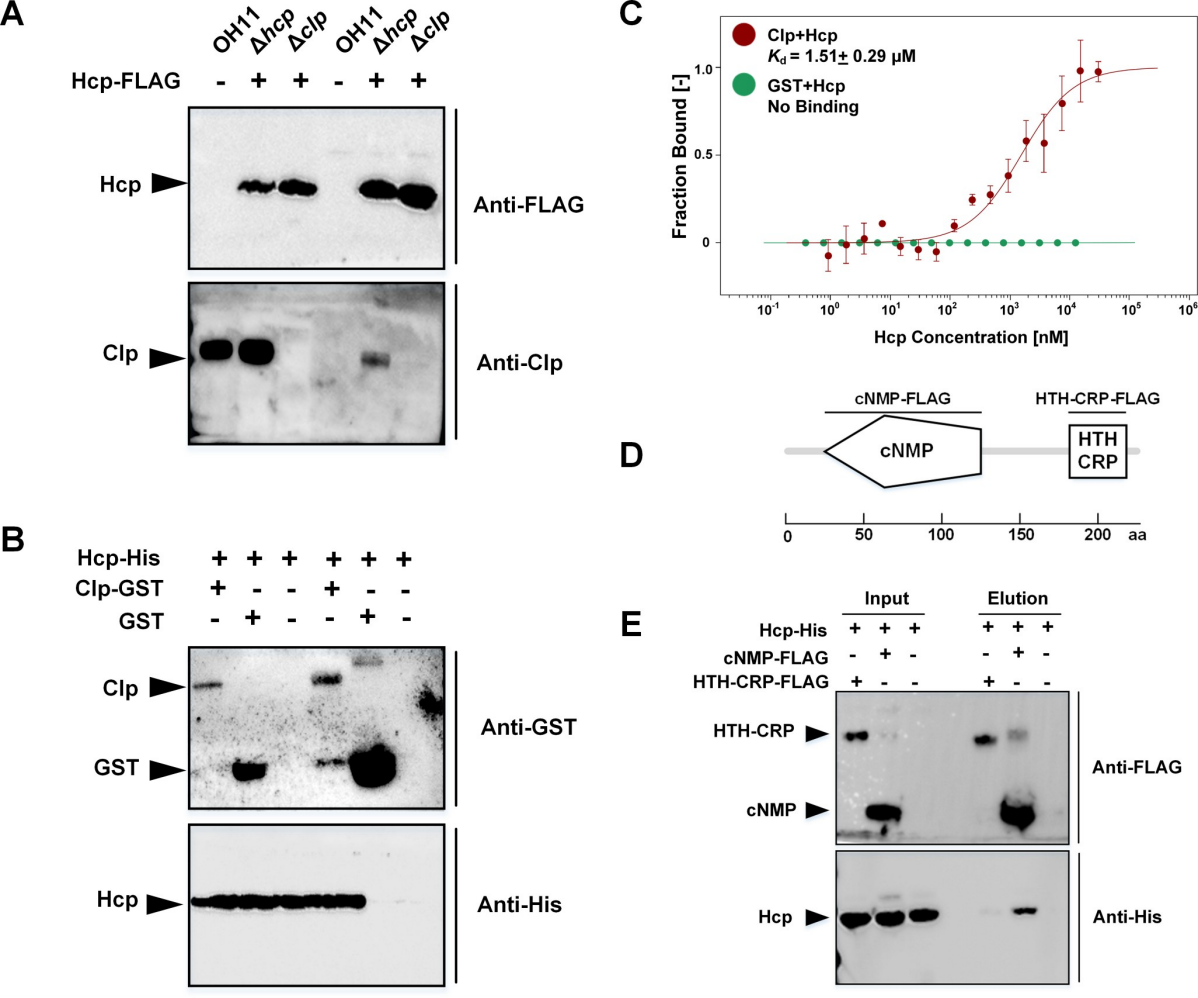
Hcp binds the transcription factor Clp. **(A)** Co-IP assays uncovering the Hcp-Clp interactions. Hcp-FLAG indicates a plasmid expressing *hcp-FLAG* in the Δ*hcp* or Δ*clp* mutants. Co-IP was carried out using cell extracts and magnetic beads coated with anti-FLAG antibodies. Proteins on the Western blot were detected by using Anti-Clp polyclonal antibody or Anti-FLAG antibody. **(B)** GST-Clp—Hcp-His_6_ pull-down assay confirming Hcp-Clp interactions. GST was served as negative control. The western blot was performed by using Anti-GST or Anti-His antibodies. **(C)** Microscale thermophoresis (MST) showing that GST-Clp binds Hcp-His_6_ (*K*_d_, 1.51 μM) (red trace), while GST does not bind Hcp-His_6_ (green trace). Constant concentration (10 μM) of the fluorescently labeled target protein (GST-Clp or GST) was titrated against increasing concentrations of unlabelled Hcp-His_6_. **(D)** A scheme showing the domain architecture of Clp comprising the cNMP-binding domain and the DNA-binding, HTH-CRP, domains. The HTH-CRP-FLAG was expressed as an N-terminal fusion to the 11-kDa Lipolyl tag (plasmid pOPTHisLip) to improve protein solubility [41]. The size of the Lipolyl-tag-HTH-CRP-FLAG is 18 kDa. The size of the cNMP-FLAG is 12 kDa **(E)** FLAG pull-down assay showing that Hcp interacts with the cNMP-binding domain of Clp.

To validate Hcp-Clp interactions, we carried out an additional co-IP assay using both the wild type and the Δ*clp* mutant. As expected, the Hcp-FLAG pulled down Clp from the extract of the wild type but not the Δ*clp* mutant (Fig 3A). Next, we performed a pull-down assay, where the glutathione S-transferase (GST) fused to Clp, GST-Clp, served as a bait to fish out Clp-interacting proteins, and a GST-specific antibody was used for precipitating protein complexes. The purified His-tagged Hcp protein, Hcp-His_6_, was captured in that assay (Fig 3B), supporting validity of the Hcp-Clp interactions.

Subsequently, we characterized the Hcp-Clp interactions using microscale thermophoresis (MST) [29]. In this assay, GST-Clp or GST were fluorescently labelled. Since Hcp-His_6_ lacked a fluorescent label, MST did not generate a signal from the Hcp self-assembly, allowing us to ignore this phenomenon. Based on the MST experiment, Hcp-His_6_ binds GST-Clp with moderate affinity (*K*_d_, 1.51 μM) (Fig 3C). Importantly, the GST-tag in itself did not bind Hcp-His_6_ (Fig 3B and 3C).

To narrow down the domain of the Clp protein is involved in Hcp binding, we expressed separately the N-terminal, so-called cNMP-binding, and the C-terminal, DNA-binding, domains of Clp (Fig 3D). The pull-down experiment revealed that Hcp binds to the N-terminal domain (Fig 3E), which further confirms specificity of Hcp-Clp interactions.

### Hcp binding lowers Clp affinity to c-di-GMP *in vitro*

To understand how Hcp-Clp binding affects HSAF biosynthesis operon expression, we considered several possibilities. First, we tested whether Hcp enhances Clp binding to DNA. An electrophoretic mobility shift assay (EMSA) confirmed that Clp binds to two sites, PA and PB, in the HSAF operon promoter (S5A Fig and S5B Fig), as described earlier [21], whereas Hcp by itself does not bind to these sites (S5C Fig). By using EMSA and MST, we tested whether or not Clp binding to the PA and PB sites is enhanced in the presence of Hcp. We found that Hcp marginally improves binding to the PA site (S5D–S5F Fig), which, in itself cannot explain the magnitude of the changes in the HSAF operon expression associated with the presence of Hcp.

**Fig 4:**
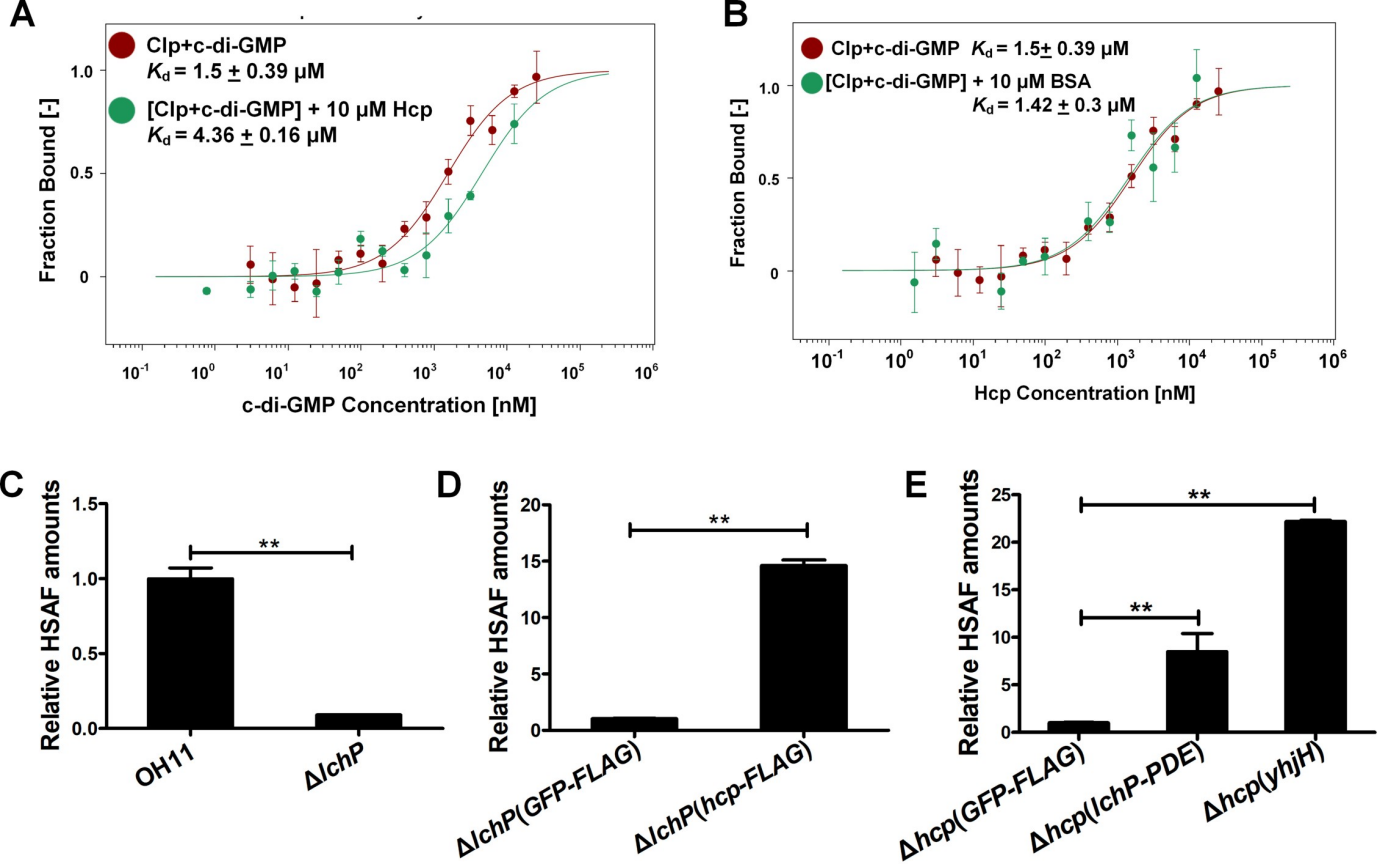
Hcp protects Clp from c-di-GMP binding, which increases HSAF production. **(A-B)** MST experiments. Fluorescently labeled proteins (10 μM) were titrated against increasing concentrations of c-di-GMP. GST-Clp—c-di-GMP binding in the absence (*K*_d_, 1.50 μM; red trace) or presence of 10 μM Hcp-His_6_ (*K*_d_, 4.36 μM; green trace) **(A)**. Lack of the effect of 10 μM BSA on GST-Clp—c-di-GMP binding (*K*_d_, 1.42 μM; green trace), and lack of Hcp-His_6_—c-di-GMP binding (No-binding; blue trace) **(B)**. **(C-D)** Relative HSAF amounts in the strains OH11 and Δ*lchP* grown in 1/10 TSB without gentamycin **(C)**, and strains containing the plasmids GFP-FLAG and Hcp-FLAG grown with gentamycin **(D)**. **(E)** HSAF production in the Δ*hcp* mutant in the presence of c-di-GMP PDEs. LchP-PDE, a cytoplasmic fragment (PAS+GGDEF+EAL domains) of the *L*. *enzymogenes* LchP; YhjH (PdeH), *E*. *coli* PDE.

Second, we tested the hypothesis that Hcp interferes with c-di-GMP binding to Clp. C-di-GMP inhibits Clp binding to the PA site in the HSAF operon promoter region, thus reducing transcription of the HSAF biosynthesis operon at high c-di-GMP levels [21]. The MST assays showed that, in the presence of Hcp-His_6_, Clp affinity to c-di-GMP was decreased by approximately three-fold (Fig 4A). Since Hcp does not bind c-di-GMP (Fig 4B), we excluded the possibility that Hcp competes with Clp for c-di-GMP binding. Further, bovine serum albumin (BSA), used as negative control, had little impact on the Clp-c-di-GMP binding (Fig 4B), suggesting that the effect of Hcp is specific. These observations indicate that Hcp binding lowers the ability of Clp to bind c-di-GMP. To validate this point, we measured if the opposite is true, i.e. if c-di-GMP can interfere with Hcp-Clp interactions. We found that increasing (5, 10, or 15 μM) levels of c-di-GMP did not affect Clp affinity to Hcp (S6 Fig). This result suggests that Hcp effectively protects Clp from c-di-GMP binding.

Lastly, we assessed the possibility that Hcp affects HSAF operon transcription by increasing Clp protein abundance, for example, by protecting Clp from proteolysis. Western blot results showed that intracellular abundance of Clp in the wild-type OH11 and the Δ*hcp* mutant was similar, at different cell densities (S7 Fig). Therefore, the hypothesis that Hcp increases Clp protein abundance had to be rejected.

### Genetic evidence that Hcp protects Clp from the inhibition by c-di-GMP

If Hcp protected Clp from c-di-GMP binding, we would expect that by increasing the level of Hcp we can mitigate the inhibitory effect of high intracellular c-di-GMP concentrations. To test this prediction we introduced the plasmid-borne *hcp-FLAG* gene in the Δ*lchP* mutant that lacks the major c-di-GMP phosphodiesterase, PDE, LchP. In the Δ*lchP* mutant, c-di-GMP concentration is elevated, Clp binding to the PA site is inhibited, and HSAF production is suppressed [21]. The plasmid-expressed Hcp-FLAG increased HSAF production in the Δ*lchP* strain by 15-fold, compared to negative control, *GFP-FLAG* (Fig 4C and 4D), consistent with our prediction. A decrease in c-di-GMP levels caused by the introduction of LchP or another, more potent, heterologous PDE, YhjH (PdeH) from *E*. *coli* [30], also resulted in the increased HSAF production (Fig 4E). These experiments support the model whereby intracellularly accumulated Hcp binds Clp, which decreases Clp affinity to c-di-GMP, and thus increases its ability to activate HSAF operon expression. Therefore, in *L*. *enzymogenes*, Hcp, when not in use as an inner tube component of the contact-dependent weapon (T6SS), assumes a new, regulatory role, whereby it stimulates production of the contact-independent weapon (HSAF).

## Discussion

**Fig 5:**
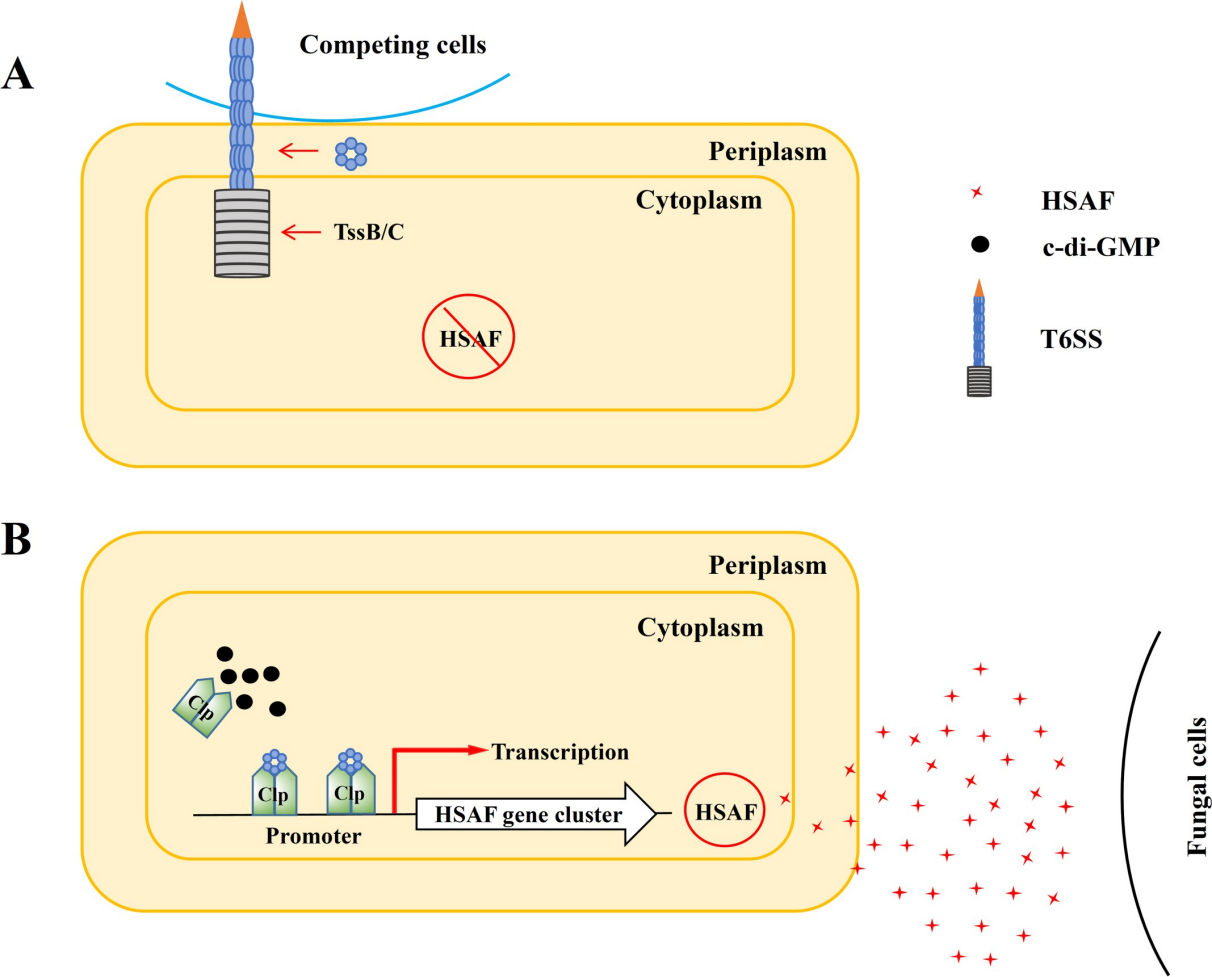
Model of coordinated production of the contact-dependent (T6SS) and contact-independent (HSAF) weapons in the soil bacterium *L*. *enzymogenes*. **(A)** In rich media (e.g., LB), which likely mimics environments crowded with microbial competitors, *L*. *enzymogenes* produces a contact-dependent weapon, T6SS. The Hcp protein makes up the inner tube, which is protected by the tail sheath made of TssB and TssC. **(B)** In nutrient-poor media (e.g., 1/10 TSB), where direct encounters with competitor cells are rare, *L*. *enzymogenes* secretes the antifungal toxin HSAF that helps fighting and preying upon filamentous oomycetes and fungi. The Hcp protein, which is not used for the T6SS assembly under these conditions, is accumulated in the cytoplasm where it protects the transcription activator Clp from the inhibition by c-di-GMP. Higher concentrations of c-di-GMP-free Clp, result in increased HSAF biosynthesis operon transcription and HSAF production.

To fend off multiple competitors in natural environments, soil bacteria like *L*. *enzymogenes* deploy multiple defense systems. The contact-dependent weapons, like T6SS, are most effective against bacterial and eukaryotic microbes whose cell walls can be penetrated by this nano-speargun. On the other hand, longer range weapons, like secreted toxin HSAF, work best against filamentous oomycetes and fungi that have thick cell walls. Since producing all weapons systems at all times can be overburdensome, bacteria rely on environmental cues to decide what systems to deploy under given circumstances. From our previous studies, we knew that *L*. *enzymogenes* deploys HSAF when grown in nutrient-poor, but not in rich media. In this study, we found out that deployment of T6SS occurs in the opposite manner, i.e. when *L*. *enzymogenes* are grown in rich media. One can envision that in natural nutrient-rich environments, density of the competing cells is high, therefore contact-dependent killing may represent a reasonable strategy (Fig 5). In the environments depleted of nutrients, where direct encounters with competitors are rare, it makes more sense to deploy contact-independent weapons, like HSAF. Further, in nutrient-poor media, *L*. *enzymogenes* is more likely to encounter filamentous oomycetes and fungi rather than other bacteria, which also supports the choice of HSAF as a weapon (Fig 5). Killing of the co-habited filamentous oomycetes and fungi gives *L*. *enzymogenes* access to the nutrients released by their prey [17, 18]. While reasonable, these predictions require verification in the natural or modelled environments, and these experiments are under way.

Our study also revealed that *L*. *enzymogenes* has evolved an intrinsic mechanism to coordinate production of contact-independent (HSAF) and contact-dependent (T6SS) weapons, which may accelerate the transition of *L*. *enzymogenes* from nutrient-rich to nutrient-poor environment. The uncovered mechanism involves accumulation of the Hcp protein in the cytoplasm, which may signal that T6SS is no longer being assembled and presumably no longer needed. We showed that cytoplasmic Hcp binds to the transcription activator of the HSAF biosynthesis operon, Clp, and that such binding lowers (by approximately 3-fold) the affinity of Clp to the inhibitory second messenger, c-di-GMP. The uninhibited, c-di-GMP-free, Clp can bind to the HSAF operon promoter, activate gene expression, and ultimately increase HSAF production (Fig 5). Importantly, our experiments suggest that Hcp is the only component of the T6SS that appears to have gained a regulatory function.

Conversely, when environmental conditions change from low to high nutrient content, the cytoplasmic Hcp is expected to be quickly recruited to the inner tube of the T6SS, which is hidden from the cytoplasm by a sheath formed by the TssB and TssC proteins. The depletion of cytoplasmic Hcp will expose Clp to inhibition by c-di-GMP and result in lower HSAF operon expression and lower production (Fig 5). These findings advance our understanding about the ways by which a soil bacterium coordinates deployment of the contact-dependent (T6SS) and contact-independent (HSAF) weapons to compete in the changing environment.

While our study has revealed interesting insights into the stimuli and mechanisms governing the selection of specific weapons by bacteria, many questions remain. While we have learned that nutrients play an important role in weapon selection in *L*. *enzymogenes*, we do not know how these nutrients are sensed and what signal transduction mechanisms operate downstream. We do not know whether intrinsic coordination of contact-independent and contact-dependent weapons is common among bacteria or unique to *L*. *enzymogenes*. Neither do we know how other weapon systems, e.g., type 4 secretion system (T4SS), are involved. *L*. *enzymogenes* does have genes for the T4SS, and the phylogenetically related species, *Xanthomonas citri* and *Stenotrophomonas maltophilia*, deploy T4SS to kill their bacterial competitors [31]. Clearly, our understanding of bacterial defense strategies remains in infancy and a lot more work is ahead.

## Materials and methods

### Bacterial strains, plasmids and growth conditions

The bacterial strains and plasmids used in this study are listed in S3 Table. Unless otherwise stated, *L*. *enzymogenes* wild type, strain OH11, and its derivative strains were cultivated in rich, Luria-Bertani (LB), medium, or in nutrient-poor, 1/10 Tryptic Soy Broth (TSB), at 28°C. Kanamycin (Km, 25 μg/mL) was added in the media for mutant construction, and gentamicin (Gm, 150 μg/mL) for plasmid maintenance. *Escherichia coli* was grown in LB medium at 37°C with appropriate antibiotics.

### Genetic methods

Double-crossover homologous recombination approaches were used to generate in-frame deletion mutants in *L*. *enzymogenes* OH11 (CGMCC No. 1978) as described previously [19]. Briefly, two flanking regions of each gene were amplified by PCR with corresponding primers and cloned into the suicide vector pEX18Gm (S3 Table). The final constructs were transformed into OH11 by electroporation. The single-crossover recombinants were selected on LB medium with 1.2% agar (LA) supplemented with Km (25 μg/mL) and Gm (150 μg/mL). The transformants were subsequently cultivated in LA plates containing 10% (w/v) sucrose and Km (100 μg/mL) to select the double crossovers. In-frame gene deletions were verified by PCR using corresponding primers listed in S4 Table. Chromosomal replacement of the native Hcp gene in the wild-type OH11 and the *tssM* mutant by a Hcp-FLAG fusion gene was also generated by the double-crossover homologous recombination approaches mentioned above.

Complementation constructs were generated as described earlier [32]. Briefly, DNA fragments containing full-length genes and their predicted promoters were amplified by PCR using primer pairs listed in S4 Table and cloned into the broad-host vector pBBR1-MCS5 (S3 Table). The plasmids were transformed into the wild-type or mutant strains by electroporation. The transformants selected on the LA plates containing Km (100 μg/mL) and Gm (150 μg/mL).

### HSAF extraction and quantification

The *L*. *enzymogenes* strains were grown in 1/10 TSB under shaking (at 220 rpm) for 24 h at 28°C. 25 mL of bacterial cultures was used for HSAF extraction as described previously [19]. Relative HSAF amounts were detected by high performance liquid chromatography (HPLC) (Agilent 1260, Santa Clara, USA) and expressed as peak intensity from HPLC chromatogram per unit of OD_600_ [19]. Three biological replicates were used, with each one analyzed in three technical replicates. Average data ± SD are shown in figures. To determine significance, we used the SPSS 14.0 package (SPSS Inc., Chicago, IL, USA) applying t-test, P = 0.05 or 0.01.

### Antimicrobial activity assays

Antimicrobial activity assay was carried out according to our previous reports [33, 34] with slight modifications. Briefly, a mycelium plug of the filamentous pathogen, *Phytophthora capsici* that was transferred from PDA (Potato Dextrose Agar) medium was inoculated on the center of 1/10 TSB agar plates. Subsequently, 2 μL of HSAF crude extracts from various *L*. *enzymogenes* strains was individually inoculated on the edge of 1/10 TSB agar plates. After two-day incubate 28°C, the antagonistic activity was monitored based on the inhibition zones around the colonies. For this, lengths of the longest axis and shortest axis of the inhibition zones were determined and averaged as the radius, according to an earlier report [33]. The inhibition zones were calculated by using the formula: area = π × (radius)^2^.

### Hcp secretion assay

The *L*. *enzymogenes* strains harboring plasmid pBBR-Hcp-FLAG were grown in 40 mL of LB and 1/10 TSB until OD_600_ reached 0.6 and 1.0, respectively. Culture supernatants were separated from cells by centrifugation at 6000 rpm at 4°C for 30 min. The supernatants were filtered through a 0.22-μM Millipore filter (Merck Millipore, Shanghai, China) and precipitated using ice-cold 10% (vol/vol) trichloroacetic acid (TCA). After an overnight incubation at 4°C, proteins were pelleted by centrifugation at 14000 rpm at 4°C for 30 min, and washed 3 times with ice-cold acetone. The resulting precipitates of secreted proteins were re-suspended in 0.01 M PBS (pH, 7.4) buffer. The concentrations of secreted proteins were measured by Thermo Scientific NanoDrop 2000. Whole cells were lysed in 2 × SDS buffer and used as positive controls. Both secreted proteins and intracellular proteins were subject to SDS-PAGE.

### Quantitative RT-PCR, qRT-PCR

qRT-PCR was performed as previously described [19]. In short, *L*. *enzymogenes* was grown in 1/10 TSB until OD_600_ reached 1.0. Total RNA was extracted from the cells using a Bacterial RNA Kit (OMEGA, Shanghai, China). The RNA samples were used as templates for cDNA synthesis using the PrimerScript RT reagent Kit with gDNA Eraser (no. RR047A, Takara, Shanghai, China). The generated cDNA samples were diluted 1:20 for qRT-PCR analysis. The primers used for qRT-PCR assays are listed in S4 Table. 16S *rRNA* gene served as an internal control. Data were analyzed by Applied Biosystems 7500 software v2.0.6. Amplification specificity was evaluated via melting curve analysis. Relative expression fold change of individual genes was calculated using the 2^−ΔΔCt^ method [35]. SPSS 14.0 (SPSS Inc., Chicago, IL, USA) was used to determine significance (*t*-test, *P* = 0.05 or 0.01).

### Expression and affinity purification of the His_6_- and GST-tagged proteins

To construct a Hcp-His_6_ fusion, the coding region of the *hcp* gene was amplified using primers listed in S4 Table and cloned into plasmid pET30a. Similarly, coding region of the cNMP-binding domain and helix-turn-helix (HTH) domain of Clp with added FLAG-tags were amplified by PCR and cloned into plasmid pET30a and pOPTHisLip, respectively (S3 Table). The constructs were transformed into *E*. *coli* BL21(DE3) (S3 Table). The His_6_-fusion proteins were purified using the Ni-NTA resin (GE Healthcare, Shanghai, China) from 500 mL *E*. *coli* BL21(DE3) cells carrying the pET30a plasmid derivatives. The strains were grown at 37°C to OD_600_ reached 0.6 and induced with 0.5 mM isopropyl β-D-1-thiogalactopyranoside (IPTG, Sigma, USA) for 4 h at 28°C. The concentration of purified proteins was determined by BCA protein assay kit (Sangon Biotech, Shanghai, China). Protein purity was assessed by sodium dodecyl sulfate polyacrylamide gel electrophoresis (SDS-PAGE). Construction, expression and purification of GST-Clp were carried out according to a recently described protocol [21].

### Co-immunoprecipitation (co-IP) and pull-down assays

Precipitation of the FLAG-tag binding proteins and their identification by mass spectrometry were performed as previously described [36]. The plasmid encoding Hcp-FLAG was introduced into the *hcp* mutant by electroporation, resulting in the Δ*hcp* (*hcp*-*FLAG*) complementation strain. This transformant was grown in 1/10 TSB until OD_600_ reached 1.0. Cells from 40 mL culture were then harvested and subsequently resuspend in 4 mL 0.01 M PBS (pH, 7.4), followed by sonication (Sonifier 250; Branson Digital Sonifier, Danbury, USA). The insoluble material was removed by centrifugation at 6000 rpm at 4°C for 30 min. 4 mL of soluble protein were mixed with 50 μL of anti-FLAG magnetic beads (Bimake, Shanghai, China) to capture Hcp interacting proteins, according to the manufacturer’s instructions. After incubation at 4°C overnight, the beads were washed three times with 500 μL of 0.01M PBS (pH, 7.4) containing 0.5% Tween 20. Proteins bound to the beads were eluted with 90 μL elution buffer (0.2 M glycine, pH 2.5), followed by eluent neutralization with 10 μL neutralization buffer (1.5 M Tris, pH 9.0). The eluted protein samples were identified by mass spectrometry at Beijing Protein Innovation Co., Ltd (Beijing, China).

Pull-down experiments involving a GST-resin were performed as described earlier [21]. The purified Hcp-His_6_ and GST-Clp proteins (5 μM each, final concentration) were added to 50 μL GST resin (GE Healthcare, Shanghai, China) in 1 mL 0.01M PBS (pH, 7.4). For a negative control, GST was used in place of GST-Clp. The mixtures tubes were incubated overnight at 4°C. The resin was collected by centrifugation and washed 6 times with 0.01 M PBS containing 1% Triton X-100 to remove non-specifically bound proteins. The proteins captured by GST-resin were eluted with a buffer containing glutathione and separated by SDS-PAGE.

For Western blots, the following antibodies where used: rabbit anti-RecA (ab63797) (Abcam, UK), polyclonal antibody to Clp (Institute of Microbiology, Chinese Academy of Sciences), His (M30111L), GST (M20007L), FLAG-tag monoclonal antibody (M20008S) (all from Abmart, Shanghai, China).

### Microscale thermophoresis, MST

The protein-c-di-GMP, protein-DNA and protein-protein interaction were assayed by microscale thermophoresis (MST) using Monolith NT.115 (NanoTemper Technologies, Germany) as described earlier [21, 37]. For the Clp-c-di-GMP and Clp-Hcp binding assays, the purified GST-Clp, GST or Hcp was labeled with the fluorescent dye NT-647-NHS (NanoTemper Technologies) via amine conjugation. Constant concentration (10 μM) of the labeled target protein in label buffer (130 mM NaHCO_3_, 50 mM NaCl) was titrated against increasing concentrations of c-di-GMP and Hcp, which were dissolved in diethylpyrocarbonate-treated water and PBS buffer, respectively. For the Clp-DNA binding assay, the constant concentration (0.5 μM) of FAM-labeled DNA probes were used against increasing concentracitons of Clp, which was dissolved in PBS buffer. The MST premium-coated capillaries (Monolith NT.115 MO-K005, Germany) were used to load the samples into the MST instrument at 25°C using 80% MST power, and 20% LED power. Fraction Bound means the baseline corrected normalized fluorescence and normalized for amplitude [38]. All experiments were performed in triplicate. Data were analyzed using Nanotemper Analysis software v.1.2.101 (NanoTemper Technologies, Germany).

### Electrophoretic mobility shift assay, EMSA

EMSA was performed as previously described [39]. The spell-out FAM-labeled probes of the 50-bp promoter region of the HSAF biosynthesis operon [21] were synthesized by GENEWIZ (Suzhou, China). The labeled DNA was mixed and incubated with various concentrations of purified proteins for 30 min on ice. The mixture was then loaded onto the 8% polyacrylamide gel and electrophoresed. EMSA signals (the labeled DNA fragments) were detected by Alex 488 nm using a VersaDoc imaging system (Bio-Rad, Philadelphia, USA).

## Supporting information

S1 Table GeneBank accession numbers of key components of T6SS. (DOC)

S2 Table Hcp-FLAG binding proteins in *L*. *enzymogenes* identified by Co-IP coupled with mass-spectrometry. (DOC)

S3 Table The bacterial strains and plasmids used in this study. (DOC)

S4 Table Primers used in this study. (DOC)

S1 Fig The effect of the FLAG-tag on Hcp on the antimicrobial activity of *L*. *enzymogenes* against *P*. *capsici*. (DOCX)

S2 Fig The effect of the *hcp* deletion on *L*. *enzymogenes* growth. (DOCX)

S3 Fig Method of HSAF measurements by HPLC. (DOCX)

S4 Fig The effects of the selected T6SS components on *L*. *enzymogenes* physiology. (DOCX)

S5 Fig The effects of Hcp on Clp binding to the promoter region of the HSAF biosynthesis operon. (DOCX)

S6 Fig The effects of c-di-GMP on Hcp-Clp binding. (DOCX)

S7 Fig The effects of Hcp on Clp abundance in *L*. *enzymogenes*. (DOCX)

S1 Reference The list of all references that are independent of the main text and cited in supplemental S1–S4 Tables and S1–S7 Figs. (DOCX)
