# Supplementary material for: An intrinsic mechanism for coordinated production of the contact-dependent and contact-independent weapon systems in a soil bacterium
Source: PLoS Pathog. 2020 Oct 9;16(10):e1008967. doi: 10.1371/journal.ppat.1008967 (PMC7577485; doi:10.1371/journal.ppat.1008967)
Supplement: S1 Table — (DOC) [file ppat.1008967.s001.doc]

**S1 Table GeneBank accession numbers of key components of T6SS**

| **GeneID** | **Gene** | **GenBank accession numbers** |
| --- | --- | --- |
| Le2326 | TssM | MT460151 |
| Le2330 | TssA | MT460152 |
| Le2340 | TssL | MT460153 |
| Le2341 | TssK | MT460154 |
| Le2342 | TssJ | MT460155 |
| Le2344 | TssB | MT460156 |
| Le2345 | TssC | MT460157 |
| Le2346 | Hcp | MT460158 |
| Le2348 | TssE | MT460159 |
| Le2349 | TssF | MT460160 |
| Le2350 | TssG | MT460161 |
| Le2351 | ClpV | MT460162 |
| Le2352 | VgrG | MT460163 |
