# Supplementary material for: An intrinsic mechanism for coordinated production of the contact-dependent and contact-independent weapon systems in a soil bacterium
Source: PLoS Pathog. 2020 Oct 9;16(10):e1008967. doi: 10.1371/journal.ppat.1008967 (PMC7577485; doi:10.1371/journal.ppat.1008967)
Supplement: S2 Table — (DOC) [file ppat.1008967.s002.doc]

**S2 Table Hcp-FLAG binding proteins in *L. enzymogenes* identified by Co-IP coupled with mass-spectrometry**

**^a^**Numbers mean the unique peptide matched with target protein in each of three independent experiments.

| **Protein** | **Predicted function** | **Numbers of Unique peptide^a^** | **Fraction of coverage^b^** |
| --- | --- | --- | --- |
| Le0413 | Ribosomal protein L14 | 6/7/4 | 26%/32%/31% |
| Le3628 | Ribosomal protein L32 | 7 | 21% |
| Le2346 | Type VI secretion system tube protein Hcp | 7/45/45 | 16%/52%/52% |
| Le2221 | hypothetical protein | 2/1 | 4%/4% |
| Le2555 | F0F1-type ATP synthase, subunit c/Archaeal/vacuolar-type H+-ATPase, subunit K | 5 |  |
| Le0848 | Clp | 4/13/10 | 10%/37%/26% |
| Le2659 | Chaperonin GroEL (HSP60 family) | 5/18/17 | 8%/29%/25% |
| Le2208 | Ribosomal protein S21 | 3/3/3 | 18%/16%/16% |
| Le0416 | Uncharacterized protein conserved in bacteria | 3/8/6 | 10%/27%/27% |
| Le1302 | L-2, 4-diaminobutyrate decarboxylase | 6 |  |
| Le2711 | TonB-dependent receptor | 5/44/40 | 0%/35%/35% |
| Le0400 | Ribosomal protein S4 and related proteins | 1/11/10 | 9%/24%/24% |
| Le3234 | Outer membrane protein and related peptidoglycan-associated (lipo)proteins | 1/8/7 | 7%/37%/24% |

**^b^** ratio of unique peptides to matched proteins from each of three independent experiments.
