# Supplementary material for: An intrinsic mechanism for coordinated production of the contact-dependent and contact-independent weapon systems in a soil bacterium
Source: PLoS Pathog. 2020 Oct 9;16(10):e1008967. doi: 10.1371/journal.ppat.1008967 (PMC7577485; doi:10.1371/journal.ppat.1008967)
Supplement: S3 Table — (DOC) [file ppat.1008967.s003.doc]

**S3 Table The bacterial strains and plasmids used in this study**

| **Strains and plasmids** | **Characteristics** | **Source** |
| --- | --- | --- |
| *Lysobacter enzymogenes* | | |
| OH11 | Wild type, Km^R^ | CGMCC no.1978 [1] |
| Δ*hcp* | In-frame deletion of *hcp*, Km^R^ | This study |
| Δ*vgrG2* | In-frame deletion of *vgrG2*, Km^R^ | This study |
| Δ*vgrG3* | In-frame deletion of *vgrG3*, Km^R^ | This study |
| Δ*clpV* | In-frame deletion of *clpV*, Km^R^ | This study |
| Δ*tssM* | In-frame deletion of *tssM*, Km^R^ | This study |
| Δ*hcp*Δ*tssM* | In-frame deletion of *tssM* under *hcp* mutant background, Km^R^ | This study |
| Δ*lchP* | In-frame deletion of *lchP*, Km^R^ | [2] |
| Δ*lafB* | In-frame deletion of *lafB*, Km^R^ | [3] |
| Δ*clp* | In-frame deletion of *clp*, Km^R^ | [4] |
| OH11-*hcp-FLAG* | The native Hcp gene was replaced at the wild-type chromosome by a Hcp-FLAG fusion gene, Km^R^ | This study |
| Δ*tssM*-*hcp-FLAG* | The native Hcp gene was replaced at the *tssM* mutant chromosome by a Hcp-FLAG fusion gene, Km^R^ | This study |
| Δ*hcp* (*GFP-FLAG*) | Δ*hcp* harboring plasmid pBBR-*GFP*-*FLAG*, Gm^R^, Km^R^ | This study |
| Δ*hcp*Δ*tssM*(*hcp-FLAG*) | Δ*hcp*Δ*tssM* double mutant harboring plasmid pBBR-*hcp*-*FLAG*, Km^R^, Gm^R^ | This study |
| Δ*hcp* (*hcp-FLAG*) | Δ*hcp* harboring plasmid pBBR-*hcp*-*FLAG*, Gm^R^, Km^R^ | This study |
| Δ*hcp* (*lchP-PDE*) | Δ*hcp* harboring plasmid pBBR-*LchP-PDE*, Gm^R^, Km^R^ | This study |
| Δ*hcp* (*yhjH*) | Δ*hcp* harboring plasmid pBBR-*yhjH*, Gm^R^, Km^R^ | This study |
| Δ*lchP* (*GFP-FLAG*) | Δ*lchP* harboring plasmid pBBR-*GFP*-*FLAG*,, Gm^R^, Km^R^ | This study |
| Δ*lchP* (*hcp-FLAG*) | Δ*lchP* harboring plasmid pBBR-*hcp*-*FLAG*, Gm^R^, Km^R^ | This study |
| OH11 (*clp-FLAG*) | OH11 harboring plasmid pBBR-*clp*-*FLAG*, Gm^R^, Km^R^ | This study |
| Δ*hcp* (*clp-FLAG*) | Δ*hcp* harboring plasmid pBBR-*clp*-*FLAG*, Gm^R^, Km^R^ | This study |
| Δ*clp* (*hcp-FLAG*) | Δ*clp* harboring plasmid pBBR-*hcp*-*FLAG*, Gm^R^, Km^R^ | This study |
| *Escherichia coli* | | |
| DH5α | Host strain for molecular cloning | [5] |
| BL21(DE3) | Host strain for protein expression | [6] |
| XL1-Blue MRF’ Kan | ∆(*mcrA*)183∆(mcrCB-hsdSMR mrr)173,*endA1,supE44,thi-1,recA1gyrA96,relA1*,lac, [F’ *proAB laclqZ*∆M15 Tn5], Km^R^ | [7] |
| Plasmids | | |
| pEX18GM | Suicide vector with a *sacB* gene, Gm^R^ | [8] |
| pEX18GM-*hcp* | pEX18GM with two flanking fragments of *hcp*, Gm^R^ | This study |
| pEX18GM-*vgrG2* | pEX18GM with two flanking fragments of *vgrG2*, Gm^R^ | This study |
| pEX18GM-*vgrG3* | pEX18GM with two flanking fragments of *vgrG3*, Gm^R^ | This study |
| pEX18GM-*clpV* | pEX18GM with two flanking fragments of *clpV*, Gm^R^ | This study |
| pEX18GM-*tssM* | pEX18GM with two flanking fragments of *tssM*, Gm^R^ | This study |
| pEX18GM-*hcp-FLAG* | pEX18GM containing the coding region of Hcp with C-terminal FLAG tag and the two flanking fragments of *hcp*, Gm^R^ | This study |
| pBBR1-MCS5 | Broad-host-range vector with a P*_lac_* promoter | [2] |
| pBBR1-*GFP*-*FLAG* | pBBR1-MCS5 containing the coding region of *GFP* with C-terminal FLAG tag and the P*_lac_* promoter | This study |
| pBBR1-*hcp*-*FLAG* | pBBR1-MCS5 containing the coding region of *hcp* with C-terminal FLAG tag and its native promoter | This study |
| pBBR1-*clp*-*FLAG* | pBBR1-MCS5 containing the coding region of *clp* with C-terminal FLAG tag and its native promoter | This study |
| pBBR1-*lchP-PDE* | pBBR1-MCS5 containing the coding region of a cytoplasmic fragment (PAS+GGDEF+EAL domains) of LchP with the P*_lac_* promoter | [2] |
| pBBR1-*yhjH* | pBBR1-MCS5 containing the coding region of *yhjH* from *E. coil* with the P*_lac_* promoter | [9] |
| pTRG | The plasmid used for protein expression in bacterial two-hybridization assay, Tet^R^ | [10] |
| pTRG-*gacS* | pTRG containing the coding region of GacS | [11] |
| pTRG-*hcp* | pTRG containing the coding region of Hcp | This study |
| pBT | The plasmid used for protein expression in bacterial two-hybridization assay, Chlo^R^ | [10] |
| pBT-*gacS* | pBT containing the coding region of GacS | [11] |
| pBT-*clp* | pBT containing the coding region of Clp | This study |
| pBT-*clp*^D52A^ | pBT containing the coding region of Clp with a D52 substitution by A52 | This study |
| pBT-*clp*^R106A&K107A^ | pBT containing the coding region of Clp with combined substitutions of R106 and K107 by A106 and A107, respectively | This study |
| pBT-*clp*^R110A^ | pBT containing the coding region of Clp with a R110 substitution by A110 | This study |
| pBT-*clp*^R122A^ | pBT containing the coding region of Clp with a R112 substitution by A112 | This study |
| pBT-*clp*^D126A^ | pBT containing the coding region of Clp with a D126 substitution by A126 | This study |
| pET30a | Protein expression vector with a His tag, Km^R^ | [6] |
| pET30a-*hcp* | pET30a containing the coding region of *hcp* | This study |
| pET30a-*cNMP-FLAG* | pET30a containing the coding region of the cNMP domain of Clp with C-terminal FLAG tag | This study |
| pGEX-6p-1 | Protein expression vector with a GST tag, Amp^R^ | [11] |
| pGEX-6p-1-*clp* | pGEX-6p-1 containing the coding region of *clp* | This study |
| pOPTHisLip | Protein expression vector with a 11-kDa Lipolyl tag, Amp^R^ | [12, 13] |
| pOPTHisLip-*HTH-CRP-FLAG* | pOPTHisLip containing the coding region of the HTH-CRP domain of Clp with C-terminal FLAG tag | This study |

^a^Km^R^, Gm^R^ Amp^R^, Tet^R^, Chlo^R^, kanamycin, gentamicin, ampicillin, tetracycline, chloramphenicol resistance, respectively.
