# Supplementary material for: An intrinsic mechanism for coordinated production of the contact-dependent and contact-independent weapon systems in a soil bacterium
Source: PLoS Pathog. 2020 Oct 9;16(10):e1008967. doi: 10.1371/journal.ppat.1008967 (PMC7577485; doi:10.1371/journal.ppat.1008967)
Supplement: S4 Table — (DOC) [file ppat.1008967.s004.doc]

**S4 Table Primers used in this study**

| **Primer** | **Sequence (5'-3')^a^** | **Purpose** |
| --- | --- | --- |
| **In-frame deletion** | | |
| *hcp* F1 | CCCAAGCTTGCATCGCCCATTACCTCAAG (*Hin*dIII) | To amplify a 474-bp fragment upstream of *hcp* |
| *hcp* R1 | GGGGTACCCAGTTCTTGGTTGCCGCTAG (*Kpn*I) |
| *hcp* F2 | GGGGTACCGCAACAGCACCTTCCAGTAC (*Kpn*I) | To amplify a 389-bp fragment downstream of *hcp* |
| *hcp* R2 | GCTCTAGAAGACCTGGCTCGGCAAGATC (*Xba*I) |
| *vgrG2* F1 | CGGGATCCGTTTCTTCTCCTTGGTGTCC (*Bam*HI) | To amplify a 525-bp fragment upstream of *vgrG2* |
| *vgrG2* R1 | CCCAAGCTTACCAACCAGGACGGCAAACC (*Hin*dIII) |
| *vgrG2* F2 | CCCAAGCTTATGTCGCGGGTGATTTCCGG (*Hin*dIII) | To amplify a 496-bp fragment downstream of *vgrG2* |
| *vgrG2* R2 | GGGGTACCCTCGCCTTCCTCGCCTTCAG (Kpn I) |
| *vgrG3* F1 | CGGGATCCCAAGTACAAGATCGCCTGAG (*Bam*HI) | To amplify a 411-bp fragment upstream of *vgrG3* |
| *vgrG3* R1 | CCCAAGCTTCATCAGGCGTTCGTGTTGGC (*Hin*dIII) |
| *vgrG3* F2 | CCCAAGCTTATGTTCTGGTTCTCGGGCTG (*Hin*dIII) | To amplify a 495-bp fragment downstream of *vgrG3* |
| *vgrG3* R2 | GGGGTACCGCACAGGAACCACGGCAATC (*Kpn*I) |
| *clpV* F1 | CCCAAGCTTAAGTGGCTGAACCTGCTGGC (*Hin*dIII) | To amplify a 378-bp fragment upstream of *clpV* |
| *clpV* R1 | GGGGTACCAGGGTCTTGAGGTTGATGCT (*Kpn*I) |
| *clpV* F2 | GGGGTACCCCAAGAGCGGGGAGTTCAAG (*Kpn*I) | To amplify a 778-bp fragment downstream of *clpV* |
| *clpV* R2 | GCTCTAGAATCAGGCGTTCGTGTTGGCG (*Xba*I) |
| *tssM* F1 | CCCAAGCTTTCAAGGCGGTGGTCAGCGAT (*Hin*dIII) | To amplify a 520-bp fragment upstream of *tssM* |
| *tssM* R1 | GGGGTACCATCCAGACCGCCAACCACAC (*Kpn*I) |
| *tssM* F2 | GGGGTACCTGAACAAGGACGACGCCACG (*Kpn*I) | To amplify a 319-bp fragment downstream of *tssM* |
| *tssM* R2 | GCTCTAGACATCAGTTGGTGGCTTTCGG (*Xba*I) |
| **Complementation** | | |
| cp*hcp* F | CGGGATCCAGTGGTAAAGGGCAGGGAGC (*Bam*HI) | To amplify a 666-bp fragment containing coding region of Hcp and its promoter region |
| cp*hcp* R | GGGGTACCTCAGGTTGCAGCGAACTGAG (*Kpn*I) |
| Chrom-*hcp-FLAG* F1 | GGGGTACCGCATCGCCCATTACCTCAAG (*Kpn*I) | To amplify a 1068-bp fragment containing the native Hcp gene with C-FLAG and two flanking fragments of *hcp* |
| Chrom-*hcp-FLAG* R1 | GGAATTCTCACTTATCGTCGTCATCCTTGTAATCGGTTGCAGCGAACTGAGGGGTGTTC (*EcoR*I) |
| Chrom-*hcp-FLAG* F2 | GGAATTCGGTTCGAGGTTGTCGGAGG (*EcoR*I) |
| Chrom-*hcp-FLAG* R2 | CCCAAGCTTCTGCCCTGGAGAAAGCCGCG (*Hin*dIII) |
| cp*hcp-FLAG* F | CGGGATCCAGTGGTAAAGGGCAGGGAGC (*Bam*HI) | To amplify a 690-bp fragment containing coding region of Hcp and its promoter region with C-terminal FLAG tag |
| cp*hcp-FLAG* R | GGAATTCTCACTTATCGTCGTCATCCTTGTAATCGGTTGCAGCGAACTGAGGGGTGTTC (*EcoR*I) |
| **qRT-PCR analysis** | | |
| qRT*- lafB* F | CATCACATCATCTCCGATGC | qRT-PCR analysis of *lafB* |
| qRT*- lafB* R | CAGTTCCACCTTCTCCTTGC |
| qRT*- pilA* F | CGTACCAGGACTACCAGGTC | qRT-PCR analysis of *pilA* |
| qRT*- pilA* R | GAAGTTGTAGGCGGTGTTGG |
| qRT-16s F | ACGGTCGCAAGACTGAAACT | An internal control for qRT-PCR |
| qRT-16s F | AAGGCACCAATCCATCTCTG |
| **Protein expression** | | |
| Hcp-His-F | GGAATTCCATATGTCTAGCGGCAACCAAGA (*Nde*I) | To amplify the Hcp coding region (531 bp) |
| Hcp-His-R | CCCAAGCTTGGTTGCAGCGAACTGAG (*Hin*dIII) |
| cNMP-FLAG-F | GGAATTCCATATGTTCCGCCCCGGCGACCCGGC (*Nde*I) | To amplify the coding region (303 bp) of the cNMP-binding domain of Clp with a C-terminal FLAG tag |
| cNMP-FLAG-R | CCCAAGCTTTCACTTATCGTCGTCATCCTTGTAATCGATCGCGTACAGCAGCTTG (*Hin*dIII) |
| HTH-CRP-FLAG-F | GGAATTCCATATGCATCCGCAGGGCACCCAGCTG (*Nde*I) | To amplify the coding region (174 bp) of the HTH DNA-binding domain of Clp with a C-terminal FLAG tag |
| HTH-CRP-FLAG-R | CCCAAGCTT﻿TCACTTATCGTCGTCATCCTTGTAATCCCCGTAGAGCACGACGGTC (*Hin*dIII) |
| **Bacterial two-hybrid** | | |
| Clp-pBT-F | CGGGATCCGTGTTCCGCCCCGGCGACCC (*Bam*HI) | To amplify the coding region (585 bp) of Clp |
| Clp-pBT-R | CCGCTCGAGTCAGCGGGTCCCGTAGAGCAC (*Xho*I) |
| Clp-pBT-F | CCAGAGGCGGCCGGATCCGTGTTCCGCCCCGGCGACCC | To amplify the coding region (585 bp) of Clp with a D52 substitution by A52 |
| Clp^D52A^-pBT-overlap-R | CACCTCGCGATGGGCGCTCTCGATGAAC |
| Clp^D52A^-pBT-overlap-F | GTTCATCGAGAGCGCCCATCGCGAGGTG |
| Clp-pBT-R | AATTCTTGCGGCCGCTCAGCGGGTCCCGTAGAGCAC |
| Clp^R106A&K107A-^pBT-overlap-F | CCTGCTCGATACCAGCGCCGCGGCCGGCCGCCTGG | To amplify the coding region (585 bp) of Clp with combined substitutions of R106 and K107 by A106 and A107, respectively |
| Clp^R106A&K107A-^pBT-overlap-R | CCAGGCGGCCGGCCGCGGCGCTGGTATCGAGCAGG |
| Clp^R110A^-pBT-overlap-F | CGCAAGGCCGGCGCCCTGGCCTTCCTCGA | To amplify the coding region (585 bp) of Clp with a R110 substitution by A110 |
| Clp^R110A^-pBT-overlap-R | TCGAGGAAGGCCAGGGCGCCGGCCTTGCG |
| Clp^R122A^-pBT-overlap-F | CCGACCGGATCGTGGCCGCCCTGCACGACCTGG | To amplify the coding region (585 bp) of Clp with a R112 substitution by A112 |
| Clp^R122A^-pBT-overlap-R | CCAGGTCGTGCAGGGCGGCCACGATCCGGTCGG |
| Clp^D126A^-pBT-overlap-F | GGTTCCTTGGCCAGGGCGTGCAGGGCGCGC | To amplify the coding region (585 bp) of Clp with a D126 substitution by A126 |
| Clp^D126A^-pBT-overlap-R | GCGCGCCCTGCACGCCCTGGCCAAGGAACC |
| Hcp-pTRG-F | CCGCTCGAGTCTAGCGGCAACCAAGAACT (*Xho*I) | To amplify the coding region (531 bp) of Hcp |
| Hcp-pTRG-R | GACTAGTTCAGGTTGCAGCGAACTGAG (*Spe*I) |
| **EMSA** | | |
| PA-F | GATATTCCAAAGAATGATCCGCGTCGCAGGATAATCGGGCCTGCGCCTTT | To amplify a 50-bp DNA probe with FAM labelling of the PA site |
| PA-R | AAAGGCGCAGGCCCGATTATCCTGCGACGCGGATCATTCTTTGGAATATC |
| PB-F | GGCGGCGGTCTCATCATCGAAACGGGGCGATGCGCTCATTTTCTTAAACA | To amplify a 50-bp DNA probe with FAM labelling of the PB site |
| PB-R | TGTTTAAGAAAATGAGCGCATCGCCCCGTTTCGATGATGAGACCGCCGCC |
