# Supplementary material for: An intrinsic mechanism for coordinated production of the contact-dependent and contact-independent weapon systems in a soil bacterium
Source: PLoS Pathog. 2020 Oct 9;16(10):e1008967. doi: 10.1371/journal.ppat.1008967 (PMC7577485; doi:10.1371/journal.ppat.1008967)
Supplement: S1 Fig — (DOCX) [file ppat.1008967.s005.docx]

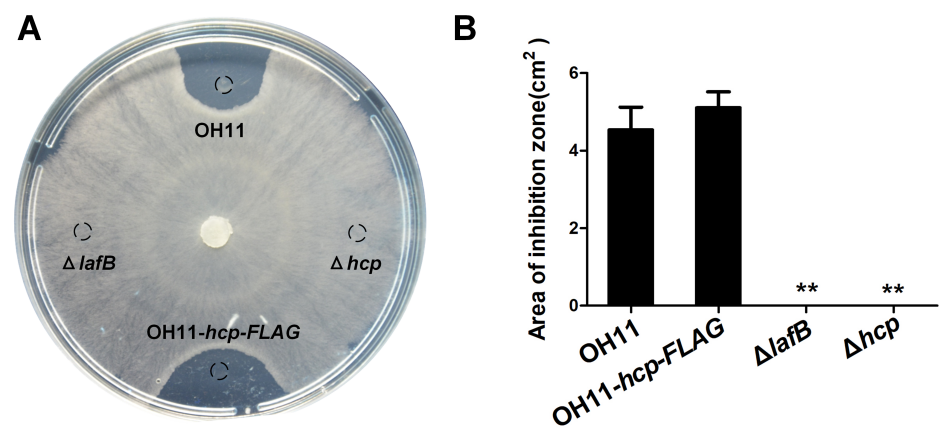


**S1 Fig. The effect of the FLAG-tag on Hcp on the antimicrobial activity of *L. enzymogenes* against *P. capsici*. (A)** Representative images showing antimicrobial activities of the HSAF crude extracts from various *L. enzymogenes* strains against *P. capsici* in 1/10 TSB agar plates. Cycles with hashed lines indicate the initial inoculation sites. **(B)** Statistical analysis of inhibition zones. Lengths of the longest axis and shortest axis of the inhibition zones were determined and averaged as the radius, R, according to an earlier report [14]. The inhibition zones were calculated by using the formula π × R^2^. Average data from three experiments are presented, ± SD. **P < 0.01 relative to the wild-type OH11. OH11, wild-type strain; Δ*hcp*, the *hcp* deletion mutant; OH11-*hcp-FLAG*, a OH11 derivative strain, in which the native Hcp gene was replaced at the wild-type chromosome by a Hcp-FLAG fusion gene; Δ*lafB*, the HSAF-deficient mutant that has an in-frame deletion in the *lafB* gene of the HSAF biosynthetic operon.
