# Supplementary figures and images for: An intrinsic mechanism for coordinated production of the contact-dependent and contact-independent weapon systems in a soil bacterium
Source: PLoS Pathog. 2020 Oct 9;16(10):e1008967. doi: 10.1371/journal.ppat.1008967 (PMC7577485; doi:10.1371/journal.ppat.1008967)

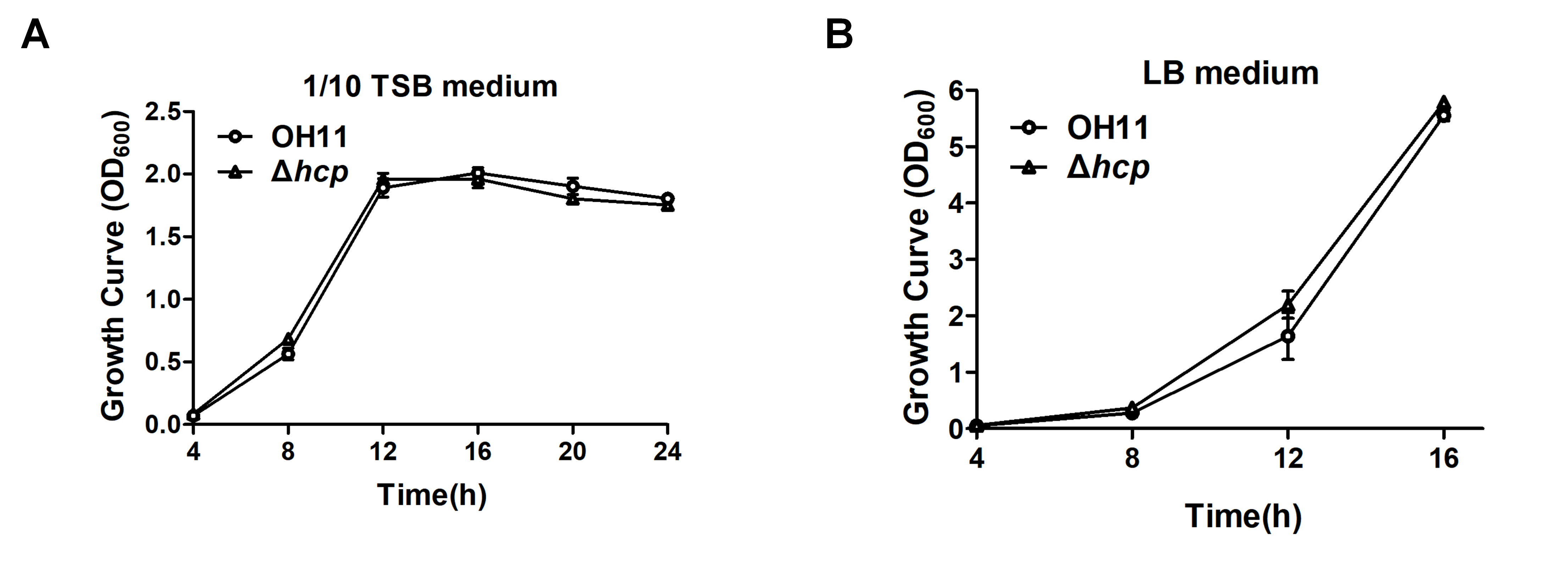


**S2 Fig. The effect of the *hcp* deletion on *L. enzymogenes* growth. (A)** 1/10 TSB. **(B)** LB.

Supplement: S2 Fig — (DOCX) [file ppat.1008967.s006.docx]
