# Supplementary material for: An intrinsic mechanism for coordinated production of the contact-dependent and contact-independent weapon systems in a soil bacterium
Source: PLoS Pathog. 2020 Oct 9;16(10):e1008967. doi: 10.1371/journal.ppat.1008967 (PMC7577485; doi:10.1371/journal.ppat.1008967)
Supplement: S3 Fig — (DOCX) [file ppat.1008967.s007.docx]

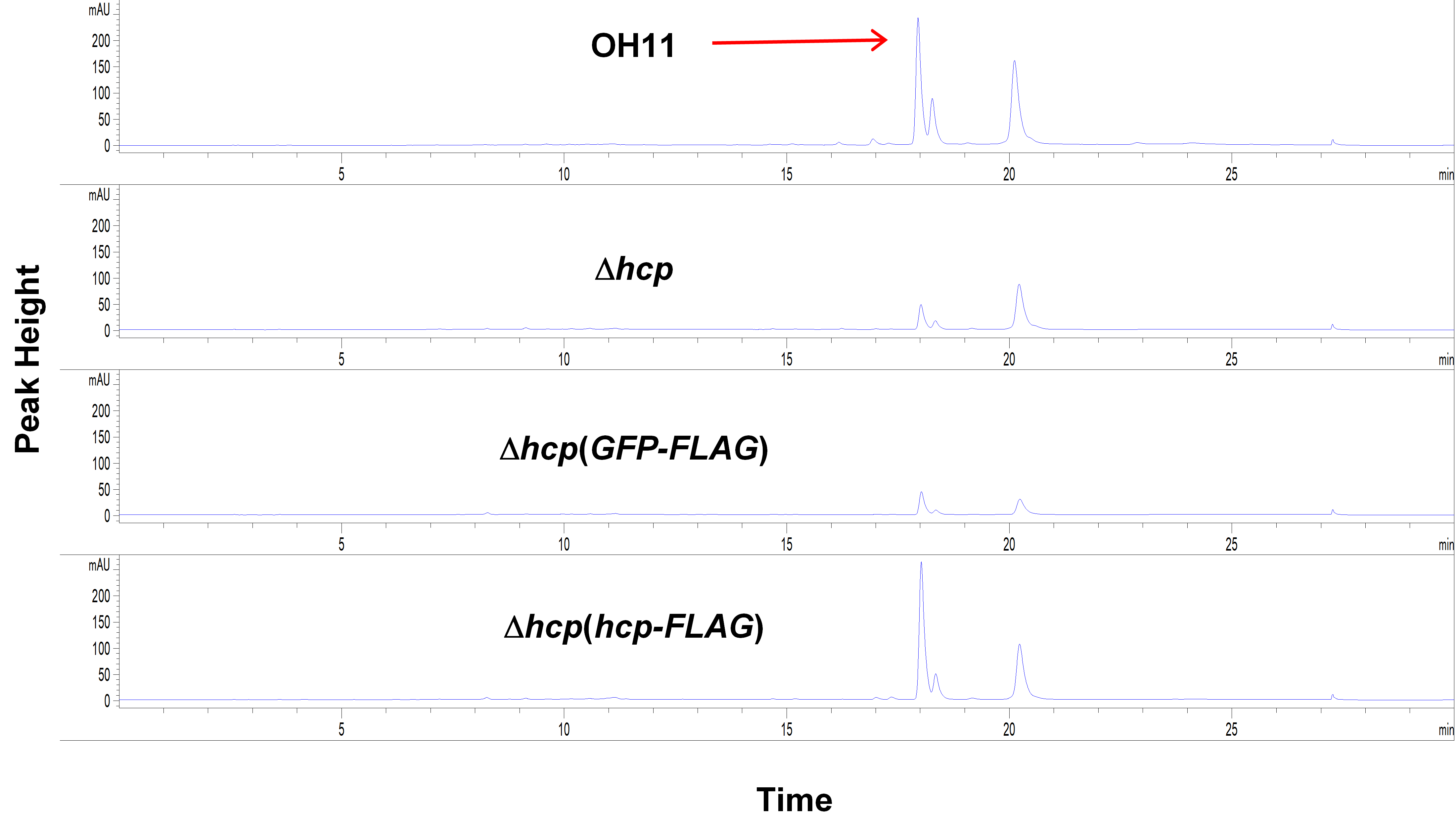


**S3 Fig. Method of HSAF measurements by HPLC.** OH11, the wild-type strain; Δ*hcp*, the *hcp* deletion mutant; Δ*hcp*(*GFP-FLAG*), the Δ*hcp* mutant complemented with a plasmid-borne GFP-FLAG gene; Δ*hcp*(*hcp-FLAG*), the Δ*hcp* mutant complemented with a plasmid-borne Hcp-FLAG gene*.* The HPLC peak showing HSAF was indicated by a red arrow.
