# Supplementary material for: An intrinsic mechanism for coordinated production of the contact-dependent and contact-independent weapon systems in a soil bacterium
Source: PLoS Pathog. 2020 Oct 9;16(10):e1008967. doi: 10.1371/journal.ppat.1008967 (PMC7577485; doi:10.1371/journal.ppat.1008967)
Supplement: S4 Fig — (DOCX) [file ppat.1008967.s008.docx]

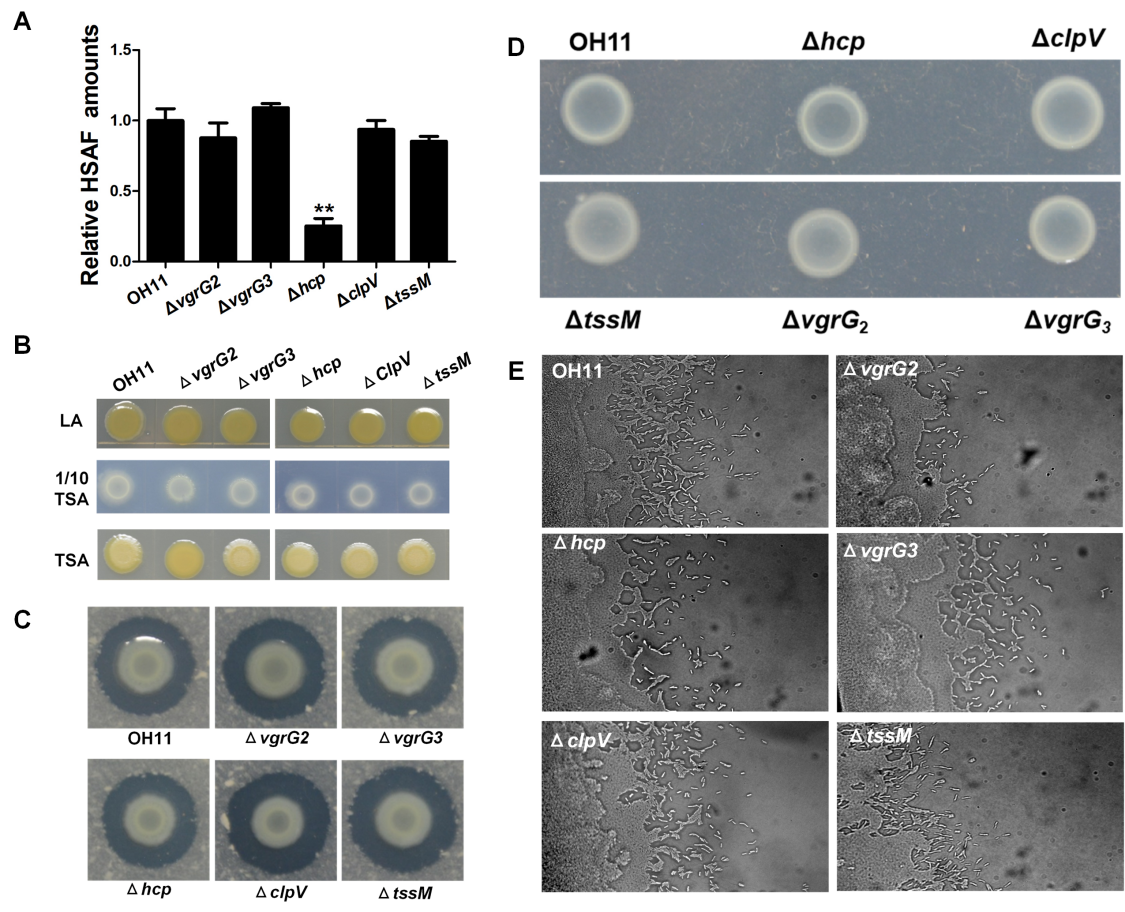


**S4 Fig. The effects of the selected T6SS components on *L. enzymogenes* physiology. (A)** HSAF levels in OH11 and T6SS gene mutants in 1/10 TSB. **(B)** Colony morphologies mutants on different nutrient media. **(C)** Extracellular chitinase production on chitin media. **(D)** Colony morphology of *L. enzymogenes* strains in 1/20 TSB agar plate. **(E)** Observation of twitching motility of *L. enzymogenes* strains in 1/20 TSA agar plate measured by microscope.
