# Supplementary material for: An intrinsic mechanism for coordinated production of the contact-dependent and contact-independent weapon systems in a soil bacterium
Source: PLoS Pathog. 2020 Oct 9;16(10):e1008967. doi: 10.1371/journal.ppat.1008967 (PMC7577485; doi:10.1371/journal.ppat.1008967)
Supplement: S5 Fig — (DOCX) [file ppat.1008967.s009.docx]

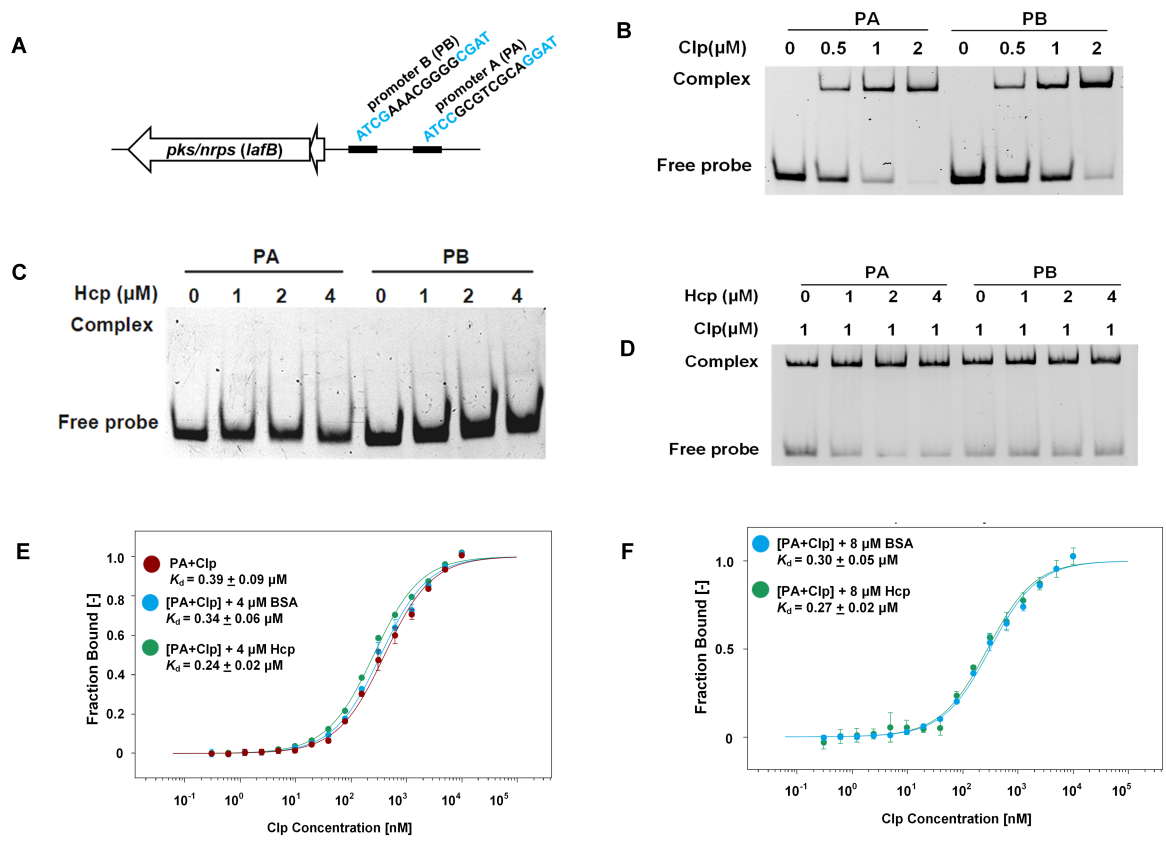


**S5 Fig. The effects of Hcp on Clp binding to the promoter region of the HSAF biosynthesis operon. (A)** Scheme of the Clp-dependent HSAF biosynthesis operon regulation [2]. PA and PB, the two Clp binding sites upstream of the *lafB* gene, the first gene in the operon [2]. **(B)** EMSA validating binding of Clp to PA and PB sites. **(C)** EMSA showing no binding of Hcp itself to PA or PB site. **(D)** EMSA showing Hcp supplement did not appear to affect the Clp-DNA (PA) complex formation. The PB site is used as a control. **(E-F)** MST further validating addition of 4-μM (**E**) or 8-μM Hcp (**F**) only has slight effects on Clp binding to the PA site. Clp binding to PA with an affinity of *K*_d_, 0.39 μM (red line). Supplement of 4-μM Hcp or 8-μM Hcp slightly altered the affinity of Clp binding to PA. BSA, a protein commonly used in biochemistry at laboratory, was used as a native control compared to that of Hcp. Constant concentration (0.5 μM) of FAM-labeled PA was used against increasing concentrations of Clp.
