# Supplementary material for: An intrinsic mechanism for coordinated production of the contact-dependent and contact-independent weapon systems in a soil bacterium
Source: PLoS Pathog. 2020 Oct 9;16(10):e1008967. doi: 10.1371/journal.ppat.1008967 (PMC7577485; doi:10.1371/journal.ppat.1008967)
Supplement: S6 Fig — (DOCX) [file ppat.1008967.s010.docx]

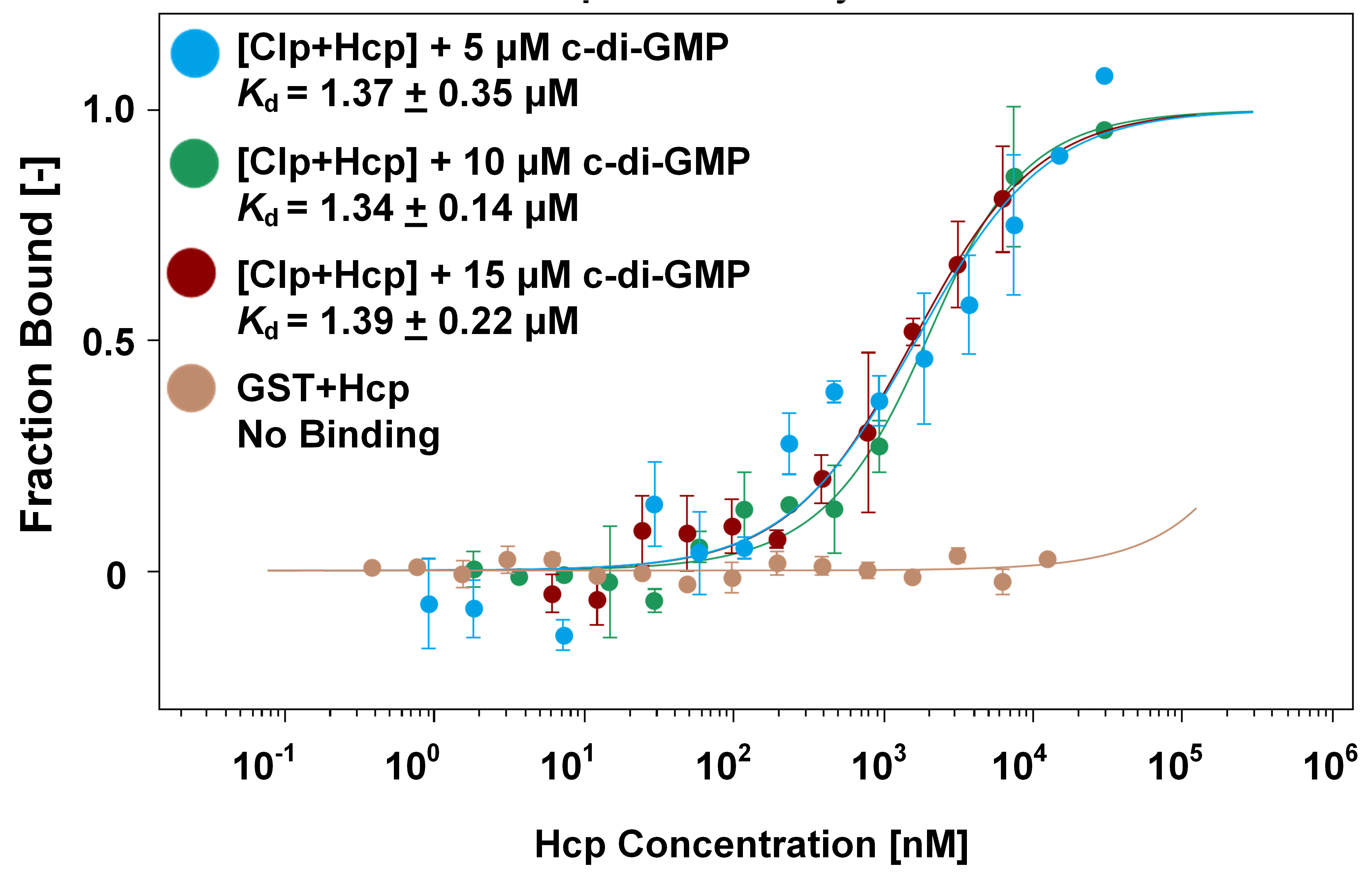


**S6 Fig. The effects of c-di-GMP on Hcp-Clp binding.** Characterization the Clp-Hcp binding affinity in the presence of c-di-GMP at final concentrations of 5 μM (*K*_d_, 1.37 μM; blue line), 10 μM (*K*_d_, 1.34 μM; green line) and 15 μM (*K*_d_, 1.39 μM; red line). GST served as negative control showing no binding with Hcp (orange line). In the absence of c-di-GMP, the Hcp-Clp binding showed an affinity of *K*_d_, 1.51 μM (**Fig. 3C**). Constant concentration (10 μM) of the GST-Clp or GST were labeled against increasing concentrations of c-di-GMP.
