# Supplementary material for: An intrinsic mechanism for coordinated production of the contact-dependent and contact-independent weapon systems in a soil bacterium
Source: PLoS Pathog. 2020 Oct 9;16(10):e1008967. doi: 10.1371/journal.ppat.1008967 (PMC7577485; doi:10.1371/journal.ppat.1008967)
Supplement: S7 Fig — (DOCX) [file ppat.1008967.s011.docx]

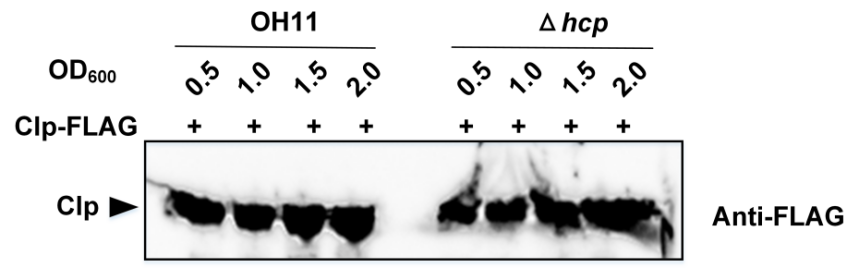


**S7 Fig. The effects of Hcp on Clp abundance in *L. enzymogenes.*** Western blot showing Hcp-FLAG levels in strains OH11 (*clp-FLAG*) and Δ*hcp* (*clp-FLAG*) grown in 1/10 TSB medium to OD_600_ of 0.5, 1.0, 1.5, 2.0, respectively. Anti-FLAG*,* monoclonal antibody against FLAG (M20008S). OH11(*hcp-FLAG*) and Δ*hcp*(*hcp-FLAG*), the wild-type OH11 and Δ*hcp* mutant complemented with a plasmid-borne *hcp-FLAG* driven by the native promoter, respectively.
