# Supplementary material for: An intrinsic mechanism for coordinated production of the contact-dependent and contact-independent weapon systems in a soil bacterium
Source: PLoS Pathog. 2020 Oct 9;16(10):e1008967. doi: 10.1371/journal.ppat.1008967 (PMC7577485; doi:10.1371/journal.ppat.1008967)
Supplement: S1 Reference — (DOCX) [file ppat.1008967.s012.docx]

**References**

1. Qian G, Hu B, Jiang Y, Liu F. Identification and characterization of *Lysobacter enzymogenes* as a biological control agent against some fungal pathogens. Agr Sci China. 2009; 8(1):68-75.
2. Xu G, Han S, Huo C, Chin KH, Chou SH, Gomelsky M, et al. Signaling specificity in the c-di-GMP-dependent network regulating antibiotic synthesis in *Lysobacter*. Nucleic Acids Res. 2018; 46(18):9276-88. http://doi.org/10.1093/nar/gky803 PMID: 30202891.
3. Wang P, Chen H, Qian G, Liu F. LetR is a TetR family transcription factor from *Lysobacter* controlling antifungal antibiotic biosynthesis. Appl Microbiol Biotechnol. 2017; 101(8):3273-82. http://doi.org/10.1007/s00253-017-8117-8 PMID: 28108764.
4. Wang YS, Zhao YX, Zhang J, Zhao YY, Shen Y, Su ZH, et al. Transcriptomic analysis reveals new regulatory roles of Clp signaling in secondary metabolite biosynthesis and surface motility in *Lysobacter enzymogenes* OH11. Appl Microbiol Biotechnol. 2014; 98(21):9009-20. http://doi.org/10.1007/s00253-014-6072-1 PMID: 25236801.
5. Qian G, Wang Y, Liu Y, Xu F, He YW, Du L, et al. *Lysobacter enzymogenes* uses two distinct cell-cell signaling systems for differential regulation of secondary-metabolite biosynthesis and colony morphology. Appl Environ Microbiol. 2013; 79(21):6604-16. http://doi.org/10.1128/AEM.01841-13 PMID: 23974132.
6. Su Z, Chen H, Wang P, Tombosa S, Du L, Han Y, et al. 4-Hydroxybenzoic acid is a diffusible factor that connects metabolic shikimate pathway to the biosynthesis of a unique antifungal metabolite in *Lysobacter enzymogenes*. Mol Microbiol. 2017; 104(1):163-78. http://doi.org/10.1111/mmi.13619 PMID: 28105648.
7. Guo M, Feng H, Zhang J, Wang W, Wang Y, Li Y, et al. Dissecting transcription regulatory pathways through a new bacterial one-hybrid reporter system. Genome Res. 2009; 19(7):1301-8. http://doi.org/10.1101/gr.086595.108 PMID: 19228590.
8. Kovach ME, Elzer PH, Hill DS, Robertson GT, Farris MA, Roop II RM, Peterson KM. Four new derivatives of the broad-host-range cloning vector pBBR1MCS, carrying different antibiotic-resistance cassettes. Gene. 1995; 166:175-6. http://doi.org/10.1016/0378-1119(95)00584-1 PMID: 8529885.
9. Chen Y, Xia J, Su Z, Xu G, Gomelsky M, Qian G and Liu F. The regulator of type IV pili synthesis, PilR, from *Lysobacter* controls antifungal antibiotic production via a c-di-GMP pathway. Appl Environ Microbiol. 2017; 83, e03397-16. http://doi.org/10.1128/AEM.03397-16 PMID: 28087536.
10. Fang X, Ahmad I, Blanka A, Schottkowski M, Cimdins A, Galperin MY, et al. GIL, a new c-di-GMP-binding protein domain involved in regulation of cellulose synthesis in enterobacteria. Mol Microbiol. 2014; 93(3):439-52. <http://doi.org/10.1111/mmi.12672> PMID: 24942809.
11. Hoang TT, Karkhoff-Schweizer RR, Kutchma AJ, Schweizer HP. A broad-host-range Flp-FRT recombination system for site-specific excision of chromosomally-located DNA sequences: application for isolation of unmarked *Pseudomonas aeruginosa* mutants. Gene. 1998; 212:77-86. http://doi.org/10.1016/s0378-1119(98)00130-9 PMID: 9661666.
12. Tan S. A modular polycistronic expression system for overexpressing protein complexes in *Escherichia coli*. Protein Expres Purif. 2001; 21(1):224-34. http://doi.org/10.1006/prep.2000.1363 PMID: 11162410.
13. Ero R, Dimitrova VT, Chen Y, Bu W, Feng S, Liu T, et al. Crystal structure of Gib2, a signal-transducing protein scaffold associated with ribosomes in *Cryptococcus neoformans*. Sci Rep. 2015; 5:8688. http://doi.org/10.1038/srep08688 PMID: 25732347.
14. Yang M, Ren S, Shen D, Chou S-H, Qian G. ClpP mediates antagonistic interaction of *Lysobacter enzymogenes* with a crop fungal pathogen. Biol Control. 2020; 140. http://doi.org/10.1016/j.biocontrol.2019.104125
